# Integration of single-cell multi-omics data by regression analysis on unpaired observations

**DOI:** 10.1186/s13059-022-02726-7

**Published:** 2022-07-19

**Authors:** Qiuyue Yuan, Zhana Duren

**Affiliations:** Center for Human Genetics and Department of Genetics and Biochemistry, Clemson University, Greenwood, SC 29646 USA

**Keywords:** Single-cell multi-omics, Regression model on unpaired observations, *Cis*-regulatory network

## Abstract

Despite recent developments, it is hard to profile all multi-omics single-cell data modalities on the same cell. Thus, huge amounts of single-cell genomics data of unpaired observations on different cells are generated. We propose a method named UnpairReg for the regression analysis on unpaired observations to integrate single-cell multi-omics data. On real and simulated data, UnpairReg provides an accurate estimation of cell gene expression where only chromatin accessibility data is available. The *cis*-regulatory network inferred from UnpairReg is highly consistent with eQTL mapping. UnpairReg improves cell type identification accuracy by joint analysis of single-cell gene expression and chromatin accessibility data.

**Supplementary Information:**

The online version contains supplementary material available at 10.1186/s13059-022-02726-7.

## Background

Recent advances in technology enable one to study heterogeneous mixtures of cell populations at the single-cell level. Single-cell RNA sequencing (scRNA-seq) [1] provides whole-genome transcription profiling, single-cell ATAC-seq (scATAC-seq) [2] identifies accessible chromatin regions, single-cell bisulfite sequencing [3] measures DNA methylation, and single-cell CUT&Tag [4] profiles histone modifications or transcription factors all at the single-cell level. Several single-cell multi-modality sequencing technologies have been developed, such as single-cell CITE-seq [5] for joint profiling of gene expression and protein expression, single-cell multiome [6] for joint profiling of gene expression and chromatin accessibility, and single-cell Paired-Tag [7] for joint profiling of gene expression and histone modification. However, it is difficult to observe all genomics profiles in the same single cell at the same time. One alternative way is to generate some modalities of genomics data on some cells and generate other modalities on other cells but from the same heterogeneous population.

Multi-omics analyses have been reported to provide a comprehensive understanding of cellular processes through the integration of different types of molecular data. Traditionally, expression and accessibility profiling are done separately on different sub-samples from the heterogeneous population. Huge amounts of such unpaired scRNA-seq and scATAC-seq, not profiled from the same cell, have been generated [8–13]. Integrative analysis of scRNA-seq with scATAC-seq could identify the subpopulations more accurately and provide more detail about the gene regulation [14–18]. For jointly analyzing these two types of data, all these methods require a linking function between *cis*-regulatory elements (REs) and target genes (TGs). For example, SOMatic [16] links the RE to the nearest gene. Our previously developed methods, Coupled NMF [18] and DC3 [17], learn the RE-TG connection from external bulk data from diverse cellular contexts and bulk 3D chromatin contact data, respectively. Distance-based linkage is problematic as some REs do not regulate the nearest genes [19]. Proper external bulk data are not always available, and the association learned from the external bulk data will omit the RE-TG relations specific to those subpopulations that have not been included in the database [15]. A more general format of the linking function is using chromatin accessibility to predict gene expression. Many methods, including Seurat [20] and Signac [21], calculate a gene activity score for scATAC-seq cells, which was defined as the read count in the gene body and promoter region. A different version of gene activity score has been defined in Cicero [22], which is a weighted sum of nearby REs where the weight is dependent on the correlation of RE and promoter accessibility; MAESTRO [15] defines gene activity score as a weighted sum of nearby REs where the weight is an exponentially decreasing function of RE-TG distances; SnapATAC [23] defines a gene accessibility score by smoothing the read count in the gene body [17–19]. An accurate linking function, RE-TG linking or a prediction of TG expression from REs, is a key to whether these methods perform well. A statistical method to learn such linking functions without using external data is in urgent need.

The main contribution of this paper is to fill this gap by the introduction of a regression model to predict the gene expression from chromatin accessibility on unpaired cells. This model allows unpaired observation of feature and response variables, say accessibility of REs are features and expression of target genes are response variables. The traditional regression model requires observation of feature variables and response variables on the same samples (we call it paired observation). Note that here we have multiple response variables and multiple feature variables. We transfer the regression problem into a covariance level quadratic equation, in which the inner products of response variables are represented as a quadratic of the inner product of feature variables. By fitting such equations, we learn the coefficient for each RE and predict gene expression of cells for which only chromatin accessibility is measured. The *cis*-regulation learned from this model would help understand the cell type-specific regulatory mechanism. The predicted gene expression from our model is a weighted sum of accessibility of gene nearby REs where the weights are the coefficients that we learned from the statistical model. Thus, this method should provide a much more accurate estimation of gene expression than previous methods. An accurate prediction of gene expression would increase the power of cell type identification and be useful for joint analysis.

## Results

### Regression analysis on unpaired observations

**Fig. 1:**
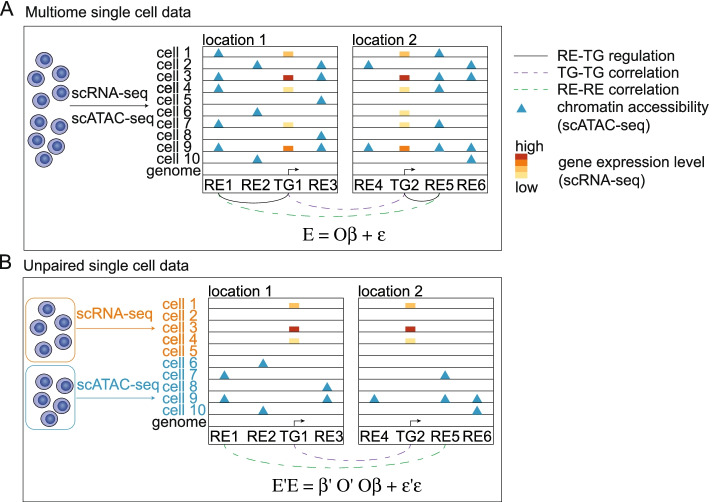
Schematic overview of UnpairReg model. **A** Schematic of the linear regression model based on paired chromatin accessibility and gene expression data. Gene expression level and chromatin accessibility are observed in the same cell. TG1 and TG2 are from two different genomic locations. RE1-TG1 regulation, RE5-TG2 regulation, TG1-TG2 correlation, and RE1-RE5 correlation are observed by data. **B** Schematic of the UnpairReg model based on unpaired data. Gene expression level is observed only for cell 1 to cell 5, while chromatin accessibility is observed for cell 6 to cell 10. TG1-TG2 correlation and RE1-RE5 correlation are observed in this data but no other significant correlation between REs and TGs. So, we infer RE1-TG1 regulation and RE5-TG2 regulation

We propose a statistical method for integrative analysis of unpaired single-cell gene expression and chromatin accessibility data from the same tissue/context but from different cells. Our method is based on a regression model on unpaired observations, so we name it unpaired regression (UnpairReg). Figure 1 shows the schematic overview of UnpairReg. Our goals are (1) to predict gene expression for cells for which only chromatin accessibility data is available and (2) to learn the *cis*-regulatory relations between regulatory elements (REs) and target genes (TGs). Let *O* be a *n*_1_ by *p*_1_ matrix representing chromatin accessibility on *p*_1_ REs for *n*_1_ cells from the first sample. The expression of TGs is noted in the *E* matrix, where *E* is a *n*_2_ by *p*_2_ matrix of gene expression data on *p*_2_ genes and *n*_2_ cells from the second sample. If we have both gene expression and chromatin accessibility data measured on the same cells (paired data), we can learn the RE-TG association by fitting a regression model *E* = *Oβ* + *ε*, where the expression of a TG is represented as a weighted sum of accessibility of REs close to this gene and the *β* represents the coefficients to be estimated (Fig. 1A). However, such a type of regression analysis is not feasible for unpaired data, where gene expression and chromatin accessibility are not measured on the same cell (Fig. 1 B).

We propose UnpairReg as a method for integrative analysis of unpaired data. First, we introduce the main idea of UnpairReg by an intuitive example in Fig. 1. Assume that we have two strongly correlated genes, TG1 and TG2, each of which has three nearby REs denoted by {RE1, RE2, RE3} and {RE4, RE5, RE6} respectively. For chromatin accessibility data, if we observe that RE1 and RE5 have a strong correlation, but the other pairs do not correlate, then we can easily infer that the regulator of TG1 is more likely to be RE1 rather than {RE2, RE3}, and the regulator of TG2 is more likely to be RE5 rather than {RE4, RE6}.

In unpaired data, the chromatin accessibility of REs is not observed for the cells on which the gene expression is available, which we call RNA-seq cells. Thus, we cannot learn the association of RE-TG by linear regression model (Fig. 1B). The remaining information, RE1-RE5 and TG1-TG2 correlations, and the information on genome location are very informative in identifying the regulation of RE to TG. Fetching information of the remaining RE-RE and TG-TG correlations from covariance matrices, UnpairReg can infer the *cis*-regulatory information and predict gene expression for the cells for which only chromatin accessibility data is available (ATAC-seq cells). Mathematically, the gene-gene covariance matrix is a quadratic of the RE-RE covariance matrix. We transfer this linear regression problem into a regression on covariance matrix under some assumption *E*^*T*^*E* = *β*^*T*^*O*^*T*^*Oβ* + *ε*^*T*^*ε*, where the *E*^*T*^*E* represents the gene-gene covariance matrix, and *O*^*T*^*O* represents the RE-RE covariance matrix. This covariance regression allows us to obtain coefficients similar to the coefficients from linear regression (see the “Methods” section for detail). We designed a fast algorithm for solving this problem by making full use of the sparse structure (i.e., REs on chromosome 1 do not regulate TGs on chromosome 2) of the coefficient matrix to iteratively update the coefficient of one gene at a time (see the “Methods” section for detail). The estimated regression coefficients reflect the *cis*-regulation, and they also can be used to predict the gene expression level for the cells for which only chromatin accessibility data is available. To further improve the prediction of gene expression, we developed an optimization model to fine-tune the predicted gene expression by preserving both cell-cell covariance and gene-gene covariance in the predicted gene expression data (see the “Methods” section for detail).

### Performance evaluation using in silico mixture of cells

To illustrate the efficacy of UnpairReg in *cis*-regulatory inference and gene expression prediction, we simulate *cis*-regulatory coefficients, gene expression, and chromatin accessibility data under different dropout rates (see Additional file 1 for detail).

**Fig. 2:**
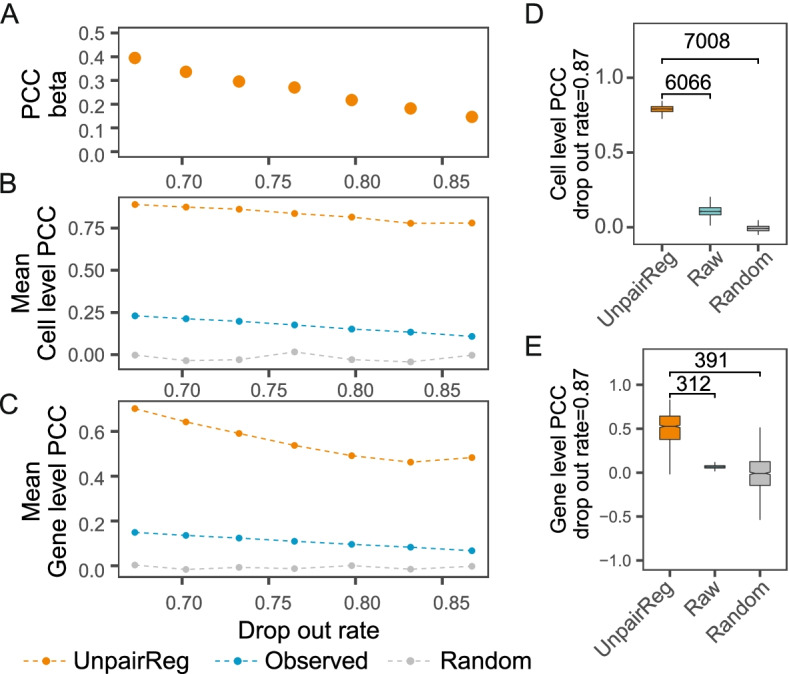
Performance of UnpairReg in silico mixture of cells. **A** PCC of UnpairReg predicted coefficient beta and real beta under different dropout rates. **B** The mean cell-level PCCs between predicted gene expression level and real gene expression. Cell-level PCC is PCC calculated for each cell across genes. Color in **B** to **E** indicates the different types of predicted gene expression data. Orange represents for UnpairReg predicted gene expression; blue represents observed gene expression after drop-out; grey represents random data. **C** The average of gene-level PCCs between predicted gene expression level and ground truth. Gene level PCC is PCC calculated for each gene across cells. **D** Cell-level PCC for each cell under a dropout rate of 0.87. The value labeled is the -log10 *p*-value of the one-sample *t*-test. **E** Gene level PCC under a dropout rate of 0.87. The value labeled is the same as **D**. Dropout rate is defined by the percentage of 0 in the single-cell data

First, we evaluate the effectiveness of the coefficient estimation. Taking the unpaired data as input, we estimate *cis*-regulatory coefficients by UnpairReg. Since we know the real *cis*-regulatory coefficient in this simulation data, we compare the estimated coefficient with the ground truth by calculating Pearson correlation coefficients (PCC) to evaluate the coefficient estimation. Figure 2A shows that the PCC ranges from 0.1 to 0.4 under a 0.6 to 0.9 dropout rate, which suggests that UnpairReg estimates the coefficient accurately.

To evaluate the performance of gene expression prediction, we take the real gene expression data as ground truth and calculate the PCC between our prediction and ground truth. Here, we calculate PCC for each cell across genes, named cell-level PCC, as well as the gene-level PCC for each gene across cells. Taking the real gene expression data as ground truth, we compare our method with the observed gene expression data. Note that the real gene expression data is different from the observed gene expression data. They both are not observed for unpaired data. The former reflects the actual level of gene expression in every single cell and cannot be observed by sequencing technologies. The latter can be measured by single-cell multiome data (paired data), and it provides a rough estimation of the gene expression as it is affected by dropout. Figure 2B shows the average cell-level PCC of our method and the observed gene expression data. The average cell-level PCC of observed gene expression decreases from 0.23 to 0.11 along with the increasing dropout rate, while the PCC of the predicted gene expression drops from 0.89 to 0.78. It shows the cell-level PCCs of UnpairReg are larger than that of observed gene expression data at each dropout rate. The average gene-level PCC of observed gene expression decreases from 0.15 to 0.07 along with the increasing dropout rate, while the PCC of the predicted gene expression decreases from 0.70 to 0.48 (Fig. 2C). Figure 2D shows the boxplot of the cell-level PCC at the 0.87 dropout rate. We compare our prediction with observed gene expression data as well as randomly generated gene expression data by one-sample *t*-test (*p*-values: 10^−6066^ and 10^−7008^). We perform the same comparison for gene-level PCC and observe a remarkable difference between our prediction and the other two predictions (*p*-values: 10^−312^ and 10^−391^, Fig. 2E).

The simulation data described above contains an equal proportion of each cell type. We generate four other simulation datasets. One of the datasets contains random numbers of cell types, and the other three include one to three cell types with a minor population (see Additional file 1 for detail). We observe similar results for the other four datasets (Additional file 1: Fig. N1 A to F). Collectively, these results suggest that UnpairReg accurately predicts the gene expression at different dropout level data and that the prediction accuracy is even more precise than the observed data.

### Predicted gene expression from UnpairReg is consistent with the multiome data

To evaluate the accuracy of UnpairReg in predicting gene expression on actual single-cell data, we apply our method to peripheral blood mononuclear cells (PBMC) and human healthy brain tissue (HHBT) multiome data from 10X Genomics (see the “Methods” section for detail). We compare UnpairReg with another gene expression prediction method named gene activity score (GAS) [21]. GAS is the method used to integrate scRNA-seq and scATAC-seq in Seurat [20], and it is defined as the number of fragments overlapping the gene body and a 2-kb upstream region for each gene. We will systematically compare UnpairReg-predicted gene expression with GAS.

**Fig. 3:**
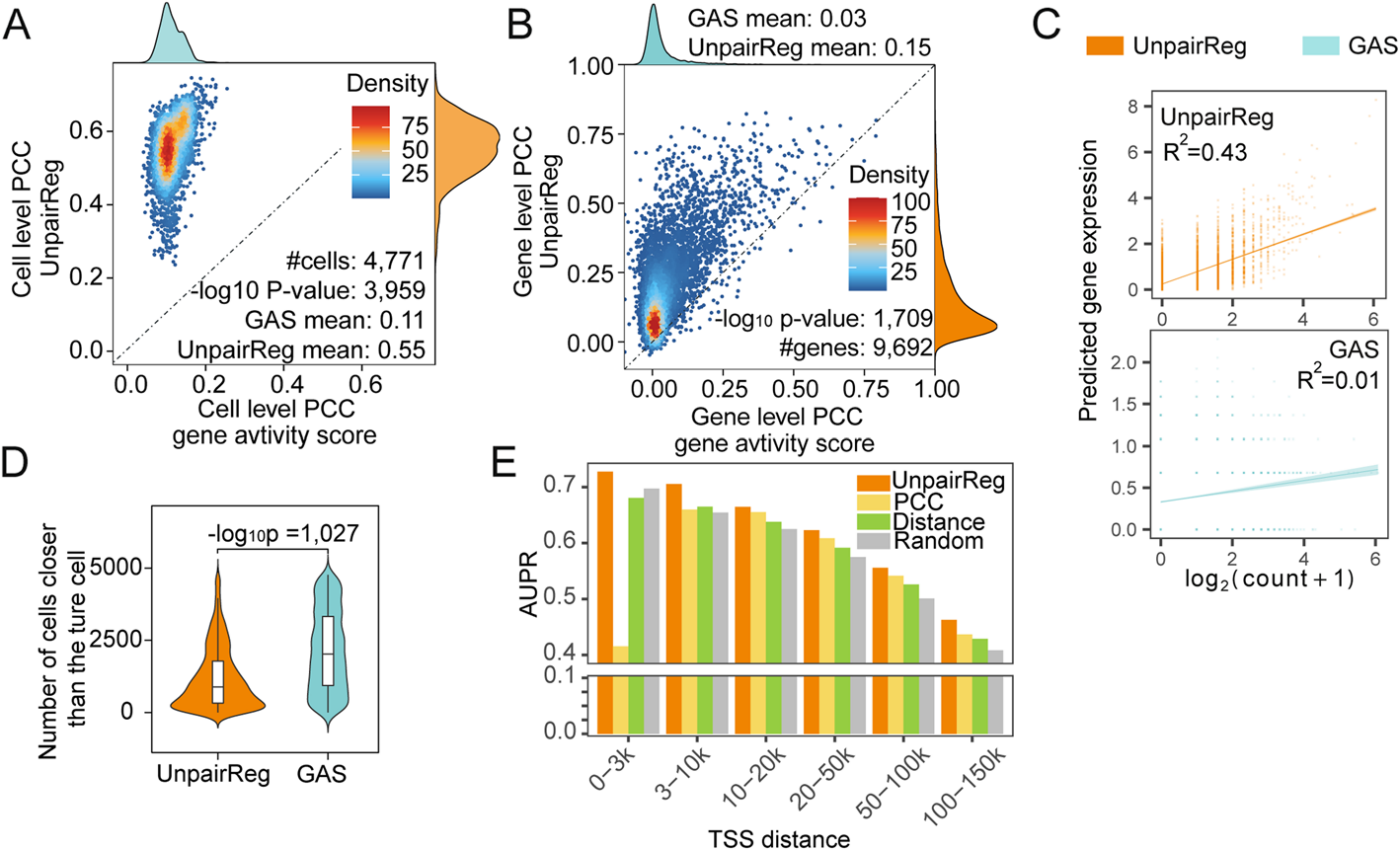
UnpairReg gene expression prediction is consistent with the paired data. **A** Cell-level PCC between the predicted and multiome gene expression. The *x*-axis represents the cell-level PCC between UnapirReg predicted gene expression and multiome gene expression, while the *y*-axis gives the cell-level PCC between GAS and multiome data. Color in **A** to **C** indicates the different methods. Orange represents for UnpairReg, and green represents GAS. **B** Gene-level PCC between predicted and multiome gene expression. The *x*-axis represents the gene-level PCC between UnapirReg predicted gene expression and multiome gene expression, while the *y*-axis represents the gene level PCC of GAS and multiome data. **C** Gene expression of multiome and UnpairReg for one gene. *R*^2^ is the *r*-squared as a goodness-of-fit measure for the linear regression model. *p*-value is for the *F*-test of linear regression. The *y*-axis and *x*-axis give predicted gene expression and log_10_ (1 + count) in a cell. **D** Alignment error of predicted gene expression. For each cell, represented by the predicted gene expression vector, we compute its distance with all cells (observed gene expression). Alignment error for a cell is defined as the number of cells that have a closer distance than the true match (the same cell). **E** The performance metrics AUPR for UnpairReg *cis*-regulatory coefficients and other methods. The ground truth is the variant-gene links from eQTLGen. We divide RE-TG pairs into different groups based on the distance of RE and the TSS of TG. There are 8634, 5791, 7431, 21,282, and 33,205 RE-TG pairs in 0–3k, 3–10k, 10–20k, 20–50k, 50–100k, and 100–150k, respectively. PCC denotes the Pearson’s correlation coefficient of RE promoter. Distance denotes the decay function of the distance to the TSS; random denotes the uniform distribution. This figure corresponds to the PMBC data

We first evaluate the accuracy of gene expression prediction for each cell by taking the gene expression count matrix as the ground truth. Figure 3A and Additional file 2: Fig S2A show the cell-level PCCs of all cells, in which the *y*-axis represents our method and the *x*-axis represents the GAS for PBMC and HHBT data, respectively. These results show that UnpairReg performs better for almost all cells (100% for PBMC and 99% for HHBT). For PBMC data, the average cell-level PCC is 0.55, which is 5-fold larger than the average PCC of GAS 0.11 (one-sample *t*-test, *p*-value 10^−3959^); for HHBT data, the average PCCs are 0.39 and 0.19 for UnpairReg and GAS (2-fold and one-sample *t*-test, *p*-value 10^−734^). Then, we compare the gene level PCC with GAS for all genes. UnpairReg achieves higher PCCs than GAS for 91.8% of genes for PBMC and 80.7% for HHBT. The average gene-level PCC for PBMC is 0.15, which is 4-fold higher than that of GAS (Fig. 3B), while the average PCC of HHBT is 0.27 (4-fold) (Additional file 2: Fig. S2C). Even though 4-fold increases the gene level PCCs, the actual PCCs of 0.15 and 0.27 cannot be considered as high correlations. We think one possible reason is that the ground truth data is affected by the dropout, and so it may not be able to reflect the real gene expression level. To duel with the dropout [24], we impute the gene expression data and then compare our prediction with the imputed gene expression data. When we take the imputed gene expression data as ground truth, cell-level PCC and gene-level PCC increase to 0.84 and 0.47 for PBMC (Additional file 2: Fig. S1 A and B), while that of HHBT increases to 0.56 and 0.53 (Additional file 2: Fig. S2 B and D). To investigate the prediction result in detail, we choose one cell and show the pattern of observed versus predicted expression (Fig. 3C). The imputation may induce bias so that these PCCs may not be accurate. The real PCCs should be in the range of PCCs calculated under raw data and PCCs calculated under imputed data.

In addition to quantifying the accuracy of gene expression prediction for each cell by correlation, we further quantify it by alignment error, defined as the number of cells that have a closer distance than the true match (see the “Methods” section for detail). Taking the gene expression count matrix as ground truth, UnpairReg achieves the lower alignment error of cells on both PBMC and HHBT datasets (Fig. 3D, one-sample *t*-test *p*-value 10^−1027^ for PBMC; Additional file 2: Fig. S2E, one-sample *t*-test *p*-value 10^−213^ for HHBT). UnpairReg achieves the lower alignment error by taking the imputed data as ground truth (Additional file 2: Fig. S1C, one-sample *t*-test *p*-value 10^−696^ for PBMC; Additional file 2: Fig. S2F, one-sample *t*-test *p*-value 10^−98^ for HHBT).

To evaluate UnpairReg more generally, we perform a systematical comparison, adding one more dataset, three more metrics, and three more methods for comparison. We apply our method to the embryonic E18 mouse brain (EEMB) (see the “Methods” section for detail). In addition to PCC as the similarity metric, we add two similarity metrics, Spearman correlation coefficient, and cosine similarity, as well as one distance, root mean square error (RMSE). Other methods to calculate GAS include Cicero [22], SnapATAC [23], and MAESTRO [15]. UnpairReg achieves higher similarity, lower distance (Additional file 2: Fig. S3 A, B, and C), and lower alignment error (Additional file 2: Fig. S3 D, E, and F) than other methods across all datasets. Overall, the predicted gene expression from UnpairReg is consistent with the single-cell multiome data and has much better performance than the predicted gene expression in previous studies.

To assess the ability of our method to infer *cis*-regulation, we calculate the consistency of the *cis*-regulatory coefficients with expression quantitative trait loci (eQTL) studies that link genotype variants to their target genes. We download variant-gene links defined by eQTL in whole blood from GTEx [25] and eQTLGen [26] and use them to validate the RE-TG prediction. As the distance between RE and TG is important for the prediction, we divide RE-TG pairs into different groups based on their distance (0–3 kb, 3–10 kb, 10–20 kb, 20–50 kb, 50–100 kb, and 100–150 kb). In each distance group, taking the eQTL data as ground truth, we computed a performance metric, area under the precision-recall curve (AUPR), by sliding the *cis*-regulatory score. We compared our method with a distance-based method, PCC between enhancer and promoter accessibility, and random predictions. UnpairReg achieves higher AUPR than other methods in all different distance groups (Fig. 3E, Additional file 2: Fig. S1D). The reason for very low AUPR of PCC in the 0–3k group is that REs in the upstream 2k of TSS are considered as promoters. These results show that UnpairReg not only can accurately predict gene expression but also can provide insight into *cis*-regulation.

### UnpairReg predicted gene expression improves the identification of cell types

**Fig. 4:**
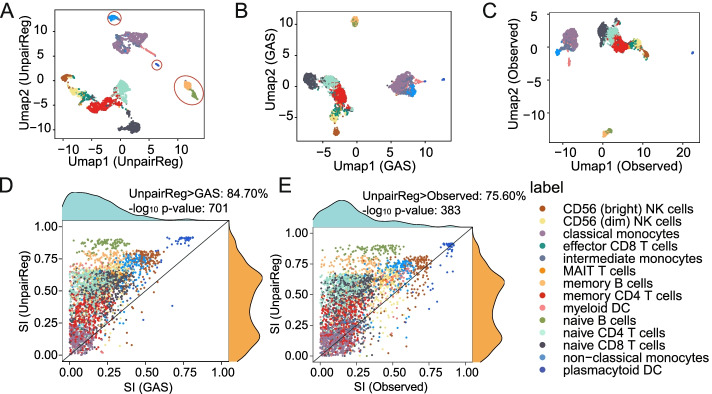
UnpairReg enhances cell type identification. **A** UMAP of the predicted gene expression from UnpairReg. Color in **A** to **E** indicates the ground truth label of PBMC data. The circled cell types from left to right are non-classical monocytes, plasmacytoid DC, memory B cells, and naïve B cells, respectively. **B** UMAP of the gene expression predicted by GAS. **C** UMAP of observed gene expression from the multiome data. **D** Silhouette index (SI) of cells based on predicted gene expression from UnpairReg and GAS. *p*-value is the significant level of the one-sample *t*-test. **E** SI of cells based on UnpairReg-predicted gene expression and the observed multiome gene expression data. *p*-value is the same as **D**

To illustrate UnpairReg’s capacity to identify the cell types or cell subpopulations, we measure whether the cluster structure embodied in the predicted gene expression data agrees with the ground truths cell type labels. Here, we use cell type annotation of PBMC data from the 10X Genomics R&D team [27] as ground truth. We compare our method with GAS and the observed gene expression data. We first perform principal component analysis (PCA) for the predicted or observed expression matrix to generate a reduced dimension matrix and choose 2nd to 20th PCs for further analysis (the first dimension tends to be highly correlated with the read depth) [9]. Figure 4A–C shows the UMAP based on the PCs of UnpairReg predicted gene expression, GAS, and observed gene expression data. We find minor populations are better separated in the UnpairReg than GAS. For example, plasmacytoid DC and non-classical monocytes. In contrast, plasmacytoid DC cells are divided into two subclusters, and non-classical monocytes are mixed with intermediate in the UMAP of GAS. We also compare our results with the observed gene expression data. Figure 4C shows that Native CD8 T cells are mixed with naive CD4 T cells in the UMAP of observed gene expression data, but they form one separate cluster in the UMAP of UnpariReg.

We further evaluate the clustering result of predicted gene expression systematically by two types of clustering analysis, single modality clustering, and coupled clustering (see the “Methods” section for detail). The first method is based on the predicted gene expression of ATAC-seq cells, and the second combines it with the gene expression of RNA-seq cells. For the single modality clustering method, we identify the cluster via the Louvain algorithm [28] based on the reduced dimension matrix (PCs). We cluster cells based on UnpairReg predicted gene expression, GAS, observed gene expression data, and impute gene expression data, respectively. We then calculate the normalized mutual information (NMI) and Adjusted Rand Index (ARI) based on the surrogate ground truth to evaluate the clusterings. Clustering based on UnpairReg predicted gene expression achieves the highest NMI and ARI (NMI 0.742 for UnpairReg, 0.582 for GAS, 0.714 for observed, and 0.724 for imputation data; ARI 0.643 for UnpairReg, 0.441 for GAS, 0.621 for observed, and 0.469 for impute). For the coupled clustering, we perform canonical correlation analysis (CCA) on the predicted gene expression of ATAC-seq cell and the observed gene expression of RNA-seq cells. Compared with the first method, NMI and ARI of UnpairReg improve to 0.791 and 0.742, respectively, while those of GAS improve to 0.700 and 0.577.

To quantify the capacity of cell type identification, we calculate the silhouette index (SI) [29] for each cell based on the top PCs (see the “Methods” section for detail). A higher SI value indicates that the cell is more similar to those sharing its label than other cell types. Figure 4D shows that UnpairReg achieves higher SI than GAS for 84.70% of cells. The mean SI of UnpairReg (0.31) is significantly higher than GAS (0.08), with a one-sample *t*-test *p*-value 10^−701^ and a fold change of 3.88. Figure 4E shows a similar result by comparing UnpairReg and observed gene expression data. UnpairReg achieves higher SI than the observed gene expression for 75.60% of cells. The average SI of UnpairReg (0.31) is 2.04-fold of the observed gene expression (0.15), and the difference is significant (one-sample *t*-test *p*-value:10^−383^).

We show the NMI and ARI of both single modality and coupled clustering, as well as the SI of 4 GAS score prediction methods in Additional file 2: Fig. S3G. UnpairReg outperforms other methods across all metrics and all methods. Together, the predicted gene expression from UnpairReg performs better in identifying distinctive cell types than the GAS and the observed gene expression data.

### UnpairReg improves co-embedding of gene expression and chromatin accessibility

Co-embedding of scRNA-seq and unpaired scATAC-seq data helps to match the cells from these two sources, identify the subpopulations, and reveal cell type-specific regulatory networks. To perform co-embedding of unpaired gene expression and chromatin accessibility data, we propose a procedure based on UnpairReg and CoupledNMF [18]. We first run UnpairReg to obtain the cis-regulatory coefficients and run CoupledNMF by taking the *cis*-regulatory coefficient as the coupling matrix (see the “Methods” section). To validate the performance of this procedure in subpopulation identification and to remove batch variation between data types, we apply our method to unpaired single-cell data from the primary bone marrow mononuclear cells (BMMC) population. The annotation of cell labels of this data is generated based on the cell type markers from bulk data analysis [30]. We compare the results with Seurat V3 co-embedding analysis [20], which links ATAC-seq cells with RNA-seq cells via canonical correlation analysis (CCA) based on GAS and gene expression data.

**Fig. 5:**
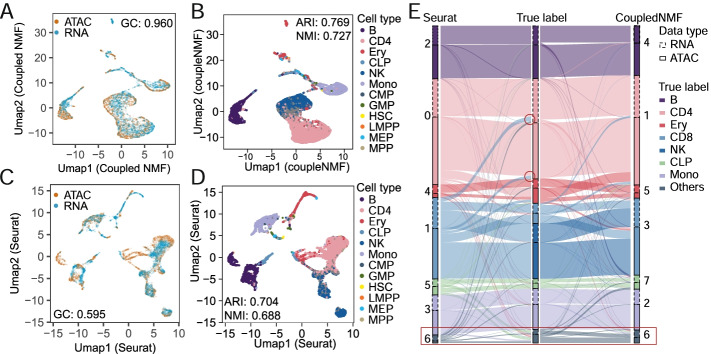
UnpairReg improves co-embedding of gene expression and chromatin accessibility. **A**, **B** UMAP of CoupledNMF co-embedding. Color in **A** and **C** indicates the data type of cells. Color in **B** and **D** indicates cell type of ground truth for the BMMC population. **C**, **D** UMAP of Seurat co-embedding. **E** Sankey plot for subpopulations of CoupledNMF, ground truth, and Seurat. Small subpopulations including Mono, HSC, CD8, GMP, and MEP are merged into the “Others” cluster

We first assess the performance of co-embedding at removing batch variation (RNA-seq and ATAC-seq) by graph connectivity (GC) [31] score, which ranges from 0 to 1 (see the “Methods” section for detail). A larger GC suggests cells of the same cell type from RNA-seq and ATAC-seq are close to one another in the co-embedding. From the co-embedding visualizations in Fig. 5A and C via UMAP and GC score, our method performs better in batch mixing.

To assess the clustering performance, we compare the clustering labels with the surrogate ground truth labels by calculating normalized mutual information (NMI) and Adjusted Rand Index (ARI) [32]. Figure 5B and D show the corresponding UMAPs colored by the surrogate ground truth labels. Our method achieves an NMI of 0.727 and ARI of 0.769, which are much higher than that of Seurat (NMI = 0.688, ARI = 0.704). To visualize the mapping of clustering labels and the ground truth, we use the Sankey plot to compare three labels: the Seurat label, ground truth labels, and our labels (Fig. 5E). To make the plot clear, we merge the 7 different progenitors’ cells (CLP, CMP, GMP, HSC, LMPP, MEP, MPP) into one cell type named progenitors cells in the ground truth since the proportion of those cells is very small and cannot be detected by current methods. Most of the CD4 cells in the surrogate ground truth map to cluster 0 and cluster 1 by Seurat and our method, respectively. In Seurat, 15.34% of CD4 RNA-seq cells are mismatched to other clusters, while this percentage in our method is 8.90%. In Addition, 67.02% of progenitor cells are mapped to cluster 6 in our method (76.47% for RNA-seq cells and 61.75% for ATAC-seq cells), while these cells are scattered in all Seurat clustering. Overall, UnpairReg identifies the clusters with higher accuracy.

## Discussion

This paper proposes a linear regression model, UnpairReg, allowing unpaired observation of feature and response variables. We apply UnpairReg to unpaired single-cell genomic data and utilize RE-RE and TG-TG correlations to infer *cis*-regulation and predict gene expression. Both simulation studies and real data analysis show that UnpairReg effectively recovers enhancer-target gene regulation and accurately predicts the gene expression. Predicted gene expression from UnpairReg is consistent with the multiome data. The inferred *cis*-regulation provides an accurate RE-TG connection for co-embedding analysis. The results show superior accuracy and robustness of UnpairReg.

From the machine learning point of view, the linear regression model using paired data to predict gene expression belongs to supervised learning, in which the feature is chromatin accessibility, and the label is gene expression. For supervised learning, features and labels are accessible for each sample. Current gene expression estimation methods, such as gene activity score, only use the chromatin accessibility data. These methods belong to unsupervised learning, in which the data is not labelled. UnpairReg falls between unsupervised and supervised learning. In detail, for each cell (sample) from scATAC-seq data, chromatin accessibility (feature) is accessible, but gene expression (label) is not accessible. For each sample in single-cell RNA-seq data, the label is accessible, but the feature is not. Taking the advantage of the input data having multiple response variables and features, UnpairReg solves this problem by building the connection between the covariance matrices of response variables and features.

At last, we discuss the limitation of our method. UnpairReg is based on the assumption that the expression of different genes is independent under the condition of REs accessibility given. But some transcription factors could regulate some target genes without changing the accessibility of REs. For those target genes, this assumption does not hold anymore, which may cause bias. The validation results reported above show that our method is already useful for many types of inference and predictions despite this. Thus, we expect that the independence assumption will not cause serious bias in the application of this method. Another limitation is that there are dropouts in the observed (raw) data. To generate data with fewer dropouts, we impute the data and use the imputation data as ground truth for several analyses. However, imputation might induce bias, although we use a naïve method to impute the raw data to avoid bias.

## Conclusions

As a linear regression-based model, UnpairReg remarkably allows unpaired observation of feature and response variables. As an integrative analysis of single-cell unpaired multi-omics data where different modalities are measured on different cells, UnpairReg yields the gene expression prediction and the *cis*-regulatory network of REs and TGs. Taking the single-cell RNA-seq data as ground truth, we compare the mean cell-level PCC and the gene-level PCC of UnpairReg and the other four methods across three datasets. The predicted gene expression from UnpairReg is about 1.67–9.93-fold (cell-level PCC) and 1.12–5.78 (gene-level PCC) fold more accurate than the widely used gene activity score, offering a good method to obtain gene expression and chromatin accessibility on the same cell. The inferred *cis*-regulatory network from UnpairReg serves as an accurate linking function of REs and TGs for a multitude of integrative analysis methods of scRNA-seq with scATAC-seq. Therefore, UnpairReg expands the opportunities in single-cell integration.

## Methods

### UnpairReg model

UnpairReg attempts to perform linear regression on the unpaired data. In the classical linear regression model, both predictors and responses are observed for each sample. We call such data paired data. Here, unpaired data allows missing predictors or responses for some samples. None of the samples has the simultaneous observation of predictor and response variables. Unpaired single-cell RNA-seq and ATAC-seq data is a good example, missing gene expression or chromatin accessibility for each cell.

We describe the UnpairReg model based on the unpaired single-cell data. Let *O* be a *n*_1_ by *p*_1_ matrix representing chromatin accessibility on *p*_1_ REs for *n*_1_ cells from the first sample. The expression of TGs is noted in the *E* matrix, where *E* is a *n*_2_ by *p*_2_ matrix of gene expression data on *p*_2_ genes and *n*_2_ cells from the second sample. For paired data, we can use the following linear regression model.

1 $$E=\ O\beta+\varepsilon$$

where *β* is the regression coefficient, and *ε* is the error term. This model does not work for unpaired data, due to the *n*_1_ cells and *n*_2_ cells being mismatched.

We transfer this linear regression problem (1) into a regression on the covariance matrix under an assumption of the expression of different genes is an independent column under the accessibility of REs given.

2 $${{E}^{T}}E={{\beta}^{T}}{O^{T}}O\beta +{{\varepsilon}^{T}}\varepsilon$$

where the *E*^*T*^*E* represents the gene-gene covariance matrix if each gene is normalized to 0 across cells, and *O*^*T*^*O* represents the RE-RE covariance matrix if each RE is normalized.

Due to the *cis*-regulation decays along with the increase of genomic distance [33], the elements of *β* which represent RE-TG regulations should also decay following the distance. Thus, we add the regularization form to penalize the long-distance regulation by an exponential transform of the distance matrix as follows.

3 $$\underset{\upbeta}{\min }{\left\Vert {E}^{T}E-{\beta}^{T}{O}^{T} O\beta \right\Vert}^{2}+\lambda {\left\Vert {A}^{\ast}\beta \right\Vert}^{2}$$

Here, ${A}_{ij}=\exp \left(\frac{d_{ij}}{d_0}\right)$ is an *p*_1_ × *p*_2_ matrix for regularizing *β*; *d*_*ij*_ is the distance between the *i*th RE and the *j*th TG (infinity for different chromosomes); * denotes Hadamard product. To avoid the spurious RE-TG regulatory on different chromosomes or far apart on the same chromosome, *β*_*ij*_ is fixed to 0 if *d*_*ij*_ is larger than a given distance *D*_0_ (200k in our study).

### Algorithm

We design a fast linear approximation algorithm for UnpairReg, taking into account the high sparsity of the *cis*-regulatory coefficient. We update *β* for one gene at a time (correspond to one column), just using the nearby REs. For one gene this step is to solve a linear ridge regression model with dozens of explanatory variables. The input of our algorithm is 4 matrices, scRNA-seq *E*, scATAC-seq *O*, *cis*-regulatory coefficient *β*_0_, and the distance between genes and REs *D*. The output is *cis*-regulatory coefficient and paired gene expression. Additionally, there are 2 tuning parameters, *λ* and *d*_0_, which have the default value of *λ* = 10^7^and *d*_0_ = 10000 in this study.

We solve β by an iteration algorithm.

Let *β* = *β*_0_, ${A}_{ij}=\exp \left(\frac{d_{ij}}{d_0}\right)$, *X* = *β*^*T*^*O*^*T*^*O*, and *Y* = *E*^*T*^*E*. Then, the quadratic equation *E*^*T*^*E* = *β*^*T*^*O*^*T*^*Oβ* can be transformed into linear equation *Y* = *Xβ*.

For each gene, we renew the corresponding column of *β*. Note *β*_*ij*_ is fixed to 0, if *d*_*ij*_ is greater than the given distance *D*_0_ (200 kbp in our study). Thus, instead of using all columns of *X* and *A*, we only retain the enhancers nearby the gene. We denoted the set of nearby enhancers of the *j*th gene as *S*_*j*_, the corresponding columns of *X* as $\hat{x}\ \left({X}_{.,{S}_j}\right)$, and corresponding rows of the *j*th gene of *A* as $\hat{a}\ \left({A}_{S_j,j}\right)$. We renew the *j*th column and the rows of nearby enhancers of *β* as ${\left({\hat{x}}^T\hat{x}+\lambda {\hat{a}}^T\hat{a}\right)}^{-1}{\hat{x}}^T{Y}_{.,j}$. Afterward, we renew the *j*th row of *X* with *β*_.*j*_ by ${X}_j.={\beta}_{.j}^T{O}^TO$.

Specify a finite number of iterations and repeat step (2) and finally obtain *β*.

Predict paired gene expression $\hat{E}= O\beta$.

### Data pre-processing

We filter genes and REs only being active in less than 1% of cells of *E* and *O* matrix, and then, we impute these two matrices for each cell by averaging *K* nearest cell (*K* is 100 in our study). To measure the distance between cells, we use PCA to generate a lower-dimensional representation of the cells and compute the Euclidean distance by only retaining the 2nd through 20th dimensions (the first dimension tends to be highly correlated with the read depth) [9]. We also denoted the gene expression and chromatin accessibility matrix after pre-processing as *E* and *O* respectively.

### Initialize cis-regulatory coefficient

To initialize the *cis*-regulatory coefficient, we sketchily estimate the gene expression matrix paired with the ATAC-seq and then compute the initialization of the *cis*-regulatory coefficient by solving (1).

We estimate gene expression ${\hat{E}}_{k_1j}$ for the cell *k*_1_ and the gene *j* by a combination of the mean expression of gene *j* and relative cell depth of *k*_1_:

4 $${\hat{E}}_{k_{1}j}=\frac{\exp\left(\frac{10}{M}\sum_{i=1}^{p_{1}}{\mathbf{1}}_{\left(\mathbf{0},+\infty\right)}\left({O}_{k_{1}i}\right)\right)}{\frac{1}{n_{1}}\sum_{k=1}^{n_{1}}\exp\left(\frac{10}{M}\sum_{i=1}^{p_{1}}{\mathbf{1}}_{\left(\mathbf{0},+\infty \right)}\left({O}_{ki}\right)\right)}\frac{1}{n_{2}}{\sum_{k=1}^{n_{2}}{E}_{kj}}$$

where $${\mathbf{1}}_{\boldsymbol{A}}\left(\boldsymbol{x}\right)=\left\{\begin{array}{l}1, if\ x\in A\\ {}0, else\end{array}\right.$$; $\sum_{i=1}^{p_1}{\mathbf{1}}_{\left(\mathbf{0},+\infty \right)}\left({O}_{k_1i}\right)$ is the number of nonzero REs in cell *k*_1_, denoting the cell depth. We transform this value to $\exp \left(\frac{10}{M}\sum_{i=1}^{p_1}{\mathbf{1}}_{\left(\mathbf{0},+\infty \right)}\left({O}_{k_1i}\right)\right)$ by an exponential transformation, also denoting a metric of cell depth; $\frac{1}{n_1}\sum_{k=1}^{n_1}\exp \left(\frac{10}{M}\sum_{i=1}^{p_1}{\mathbf{1}}_{\left(\mathbf{0},+\infty \right)}\left({O}_{ki}\right)\right)$ denotes the mean cell depth. Therefore, $\frac{\exp \left(\frac{10}{M}\sum_{i=1}^{p_1}{\mathbf{1}}_{\left(\mathbf{0},+\infty \right)}\left({O}_{k_1i}\right)\right)}{\frac{1}{n_1}\sum_{k=1}^{n_1}\exp \left(\frac{10}{M}\sum_{i=1}^{p_1}{\mathbf{1}}_{\left(\mathbf{0},+\infty \right)}\left({O}_{ki}\right)\right)}$ is a fold change, denoting the relative cell depth of cell *k*_1_; $\frac{1}{n_2}\sum_{k=1}^{n_2}{E}_{kj}$ denotes the mean expression of the cell *j*. The rationale here is combining the mean expression level for each gene from RNA-seq data and the expression level for each cell (cell depth) from ATAC-seq data to predict gene expression in ATAC-seq data. Then, we perform linear regression based on the predicted gene expression and the imputed chromatin accessibility matrix *O*, and generate *β*_0_ as the initial value of the regulatory matrix. *β*_0_ is given by ${\beta}_0={\left({O}^TO\right)}^{-1}{(O)}^T\hat{E}$.

### Fine-tuning of gene expression

We have gained the predicted gene expression from the algorithm above based on the gene-gene covariance and RE-RE covariance. To improve the gene expression in preserving both cell-cell covariance and gene-gene covariance better, we developed an optimization model to fine-tune the predicted gene expression further. We design an optimization model to find a fine-tuned gene expression X, so that the predicted gene-gene covariance (*X*^*T*^*X*) is the same with the real gene-gene covariance (*R*_1_), and meanwhile, the predicted RE-RE covariance (*XX*^*T*^) is the same with the real one:

5 $$\underset{\mathrm{X}}{\min}\left\Vert {R}_1-{X}^TX\right\Vert {}_F{}^2+{\lambda}_1\left\Vert {R}_2-{XX}^T\right\Vert {}_F{}^2$$

Here, ${\left\Vert {R}_1-{X}^TX\right\Vert}_F^2$ aims to preserve gene-gene covariance, where ${R}_1=\frac{n_1}{n_2}{E}^TE$ is the gene-gene covariance, defined by *E*^*T*^*E* timing a scaling factor, the ratio of the number of cells, to eliminate the gap of cell number between RNA-seq and ATAC-seq, matching the scale of *E*^*T*^*E* and *X*^*T*^*X*. ${\left\Vert {R}_2-X{X}^T\right\Vert}_F^2$ aims to preserve cell-cell covariance, where ${R}_2=\frac{\mu_2}{\mu 1}O{O}^T$ is the cell-cell covariance, defined by *OO*^*T*^ timing another scaling factor the ratio of the mean of *EE*^*T*^ and *OO*^*T*^ to eliminate the difference of data type. *λ*_1_ is a tuning parameter to weight the two objectives. The scaling factor is introduced. We design a fixed point iterative algorithm, taking $\hat{E}= O\beta$ as the start point, renewing *X* by ${X}_{ij}={X}_{ij}\frac{B_{ij}}{C_{ij}}$, where *B* = *XR*_1_ + *λ*_1_*R*_2_*X* and *C* = (1 + *λ*_1_)*XX*^*T*^*X*.

### Simulation

To illustrate the efficacy of UnpairReg in regulatory inference and gene expression prediction, we performed a simulation study. We simulate the scATAC-seq data according to the method proposed in ref [34] taking bulk ATAC-seq count matrix [35] as input. We use Lun [36] to simulate scRNA-seq data. The detail about the simulation pipeline is described in the Additional file 1.

### PBMC 10x data

We download the PBMC 10K data from the 10X genomics website https://support.10xgenomics.com/single-cell-multiome-atac-gex/datasets. Note that it contains 11,909 cells, and the granulocytes were removed by cell sorting of this dataset. We use the filtered cells by features matrix from the output of 10X genomics software Cell Ranger ARC as input and perform the downstream analysis. First, we perform Seurat 4.0 [37] weighted nearest neighbor (WNN) analysis and it removes 1497 cells. We also remove the cells that do not have surrogate ground truth and it results in 9543 cells. Then, we generate the unpaired data by randomly selecting 4771 cells as ATAC-seq data with the remaining 4772 cells as RNA-seq data. We next perform the UnpairReg procedure, remaining 33,050 peaks and 11,277 genes when preprocessing data, only considering the REs within 200 kbp from TSS. The ground truth of predicted gene expression is the RNA-seq data of the 4771 cells.

### HHBT 10x data

We download the HHTB data from the 10X genomics website https://support.10xgenomics.com/single-cell-multiome-atac-gex/datasets. We use the filtered cells by features matrix from the output of the 10X genomics software Cell Ranger ARC as input and perform the downstream analysis. It contains 3332 cells. We generate unpaired data by taking all cells as RNA-seq cells and ATAC-seq cells. We next perform the UnpairReg procedure, remaining 48,053 peaks and 15,980 genes when preprocessing data, only considering the REs within 200 kbp from TSS. The ground truth of predicted gene expression is the RNA-seq data of the 3332 cells.

### EEMB 10x data

We download the EEMB data from the 10x genomics website https://support.10xgenomics.com/single-cell-multiome-atac-gex/datasets. We use the same pipeline to analyze the data, generating scRNA-seq data with 15,109 genes and 4881 cells, as well as scATAC-seq data with 37,758 peaks and 4881 cells.

### Clustering of cells

We identify the cluster by 2 methods. For the first method, we perform PCA based on the gene expression data and nomalize each PC, rescaling the standard deviation to 1. Then, we perform Louvain algrithm based on the 2nd to the 20th PCs. In the second method, we perform canonical correlation analysis (CCA) taking predicted gene expression data of ATAC-seq cells and the gene expression data of RNA-seq cells as input. The output of CCA is a co-embedding of ATAC-seq and RNA-seq cells. We cluster the cells via Louvain algorithm based on the co-embedding.

### Alignment error

Alignment error is used to evaluate the alignment accuracy of each cell. Denote *X* and *Y* as the predicted gene expression matrix and ground truth of gene expression. The alignment error for the *k*th cell is defined as:

6 $${A}_k={n}_1^{(k)}+{n}_2^{(k)}$$

7 $${\displaystyle \begin{array}{c}{n}_1^k=\sum_{i=1}^K{1}_{d\left({x}_{k.},{y}_{i.}\right)<d\left({x}_{k.},{y}_{k.}\right)}\left(d\left({x}_{k.},{y}_{i.}\right),d\left({x}_{k.},{y}_{k.}\right)\right)\end{array}}$$

8 $${\displaystyle \begin{array}{c}{n}_2^k=\sum_{i=1}^K{1}_{d\left({x}_{i.},{y}_{k.}\right)<d\left({x}_{k.},{y}_{k.}\right)}\left(d\left({x}_{i.},{y}_{k.}\right),d\left({x}_{k.},{y}_{k.}\right)\right)\end{array}}$$

where ${n}_1^k$ is the number of cells which is closer to the *k*th cell than the same cell in the ground truth gene expression data, *d* is the Euclidean distance, and *x*_*k*._ *and y*_*k*_. represent the predicted and ground truth gene expression of the *k*th cell, respectively.

### SI

Based on the 2nd to the 20th PCs obtained from the first clustering method, we compute SI [29] for each cell using cosine distance.

### Co-embedding analysis for BMMC population

BMMC data is unpaired single-cell data with 2760 cells from scRNA-seq and 3928 cells from scATAC-seq (GEO under accession number GSE159417). After we get regulatory matrix *β* , we perform CoupledNMF [18] replacing the *A* matrix in (3) by *β*. *H*_1_ and *H*_2_ give the cluster membership (7 clusters) of ATAC-seq and RNA-seq cells. We perform CCA for co-embedding by finding linear combinations of *H*_1_ and *H*_2_ which have a maximum correlation with each other. We get a co-embedding matrix via CCA, the embedding of scRNA-seq and scATAC-seq cells, for further analysis, including UMAP and computing graph connectivity score.

The optimization problem for CoupledNMF is as follows:

9 $${\displaystyle \begin{array}{c}{\min_{W_1,{H}_1,{W}_2,{H}_2\ge 0}} \frac{1}{2}{\parallel O-{W}_1{H}_1\parallel}_F^2+\frac{\lambda_1}{2}{\parallel E-{W}_2{H}_2\parallel}_F^2-{\lambda}_2\mathrm{tr}\left({W}_2^TA{W}_1\right)+\mu \left({\parallel {W}_1\parallel}_F^2+{\parallel {W}_2\parallel}_F^2\right)\end{array}}$$

Seurat co-embedding analysis follows the “integrating scRNA-seq and scATAC-seq data” vignette (https://satijalab.org/seurat/articles/atacseq_integration_vignette.html).

### Graph connectivity

The graph connectivity metric assesses whether cells of the same type from different batches (RNA and ATAC in our study) are close to one another in the embedding. This is evaluated by computing a *k*-nearest neighbor (kNN) graph, *G*(*N*; *E*), on the co-embedding using Euclidean distances. We then check if all cells with the same cell type label are connected on this kNN graph. For each cell type label *c*, we generate the subset kNN graph *G*(*N*_*c*_; *E*_*c*_), which contains only cells from a given label. Using these subset kNN graphs, we compute the graph connectivity score:

10 $${\displaystyle \begin{array}{c} gc=\frac{1}{\left|C\right|}\sum_{c\in C}\kern0.1em \frac{\left| LCC\left(G\left({N}_c;{E}_c\right)\right)\right|}{\left|{N}_c\right|}\end{array}}$$

Here, *C* represents the set of cell type labels, |*LCC*(*G*(*N*_*c*_; *E*_*c*_))| is the number of nodes in the largest connected component of the graph, and |*N*_*c*_| is the number of nodes with cell type *c*. The resulting score has a range of (0, 1], where 1 indicates that all cells with the same cell type are connected in the integrated kNN graph.

### Comparison of PCCs from two different methods

We use a one-sample *t*-test to compare the two groups because the samples are a sample of the two groups. So, we test whether the mean difference of the two group is different from zero, which is a one-sample *t*-test.

### Model parameters

We use the default parameters of UnpairReg for all analyses in this manuscript. We set the fixed distance *d*_0_ in matrix A from (3) 10,000 as the default value. The tuning parameters in the *λ* in (3) and *λ*_1_ in (5) are set as 10^7^ and 0.5, respectively. We use default parameters for methods for comparison, including Signac, Cicero, MAESTRO, and snapATAC. For Signac, we use the GeneActivity function from Signac R package 1.4.1 to get the gene activity score following the tutorial on Signac website https://satijalab.org/signac/articles/pbmc_vignette.html. For Cicero, we calculate the score by normalize_gene_activities function implemented in the Cicero R package 1.3.5. We follow the recommended analysis protocol https://cole-trapnell-lab.github.io/cicero-release/docs_m3/ from the Cicero website. For MAESTRO, we perform the calculate_RP_score function from MAESTRO R package 1.5.1. For snapATAC, we take the standard pipeline, analyzing the 5k PBMC dataset from 10X genomics https://kzhang.org/SnapATAC2/tutorials/pbmc.html, as reference. The version of the SNAPATAC2 python module is 1.99.99.3. The gene activity score is calculated by the snap.pp.make_gene_matrix function.

## Supplementary Information

**Additional file 1:.** The simulation and algorithm details include simulation pipeline, simulation result, algorithm performance, and figures.

**Additional file 2: Fig S1.** UnpairReg gene expression prediction is consistent with the paired data. **Fig S2.** UnpairReg gene expression prediction of HHBT is consistent with the paired data. **Fig S3.** Systematical evaluating the performance of UnpairReg. (A) to (C) The mean gene level and cell level similarity/distance of predicted gene expression and raw data. RMSE is scaled by dividing by the maximum of 5 methods.

**Additional file 3..** Review history.

## Data Availability

The multiome data (PBMC and HHBT) used during this study are downloaded from the 10X Genomics website [38]. BMMC data is downloaded from the NCBI Gene Expression Omnibus (GEO, https://www.ncbi.nlm.nih.gov/geo/) under accession number GSE159417 [30]. The software is available at GitHub at https://github.com/Durenlab/UnpairReg [39] and the Zenodo repository under the GPLv3 license [40].
