# Supplementary material for: Integration of single-cell multi-omics data by regression analysis on unpaired observations
Source: Genome Biol. 2022 Jul 19;23:160. doi: 10.1186/s13059-022-02726-7 (PMC9295346; doi:10.1186/s13059-022-02726-7)
Supplement: Supplementary file 1 — Additional file 1:. The simulation and algorithm details include simulation pipeline, simulation result, algorithm performance, and figures. [file 13059_2022_2726_MOESM1_ESM.pdf]

Supplementary Note for

Integration of single cell multi-omics data by regression analysis on unpaired observations

Qiuyue Yuan[1] and Zhana Duren[1,#]
[1] Center for Human Genetics and Department of Genetics and Biochemistry, Clemson University, Greenwood, SC 29646, USA
# Corresponding Author: zduren@clemson.edu

This PDF file includes:

Simulation pipeline
Simulation result
Algorithm performance
Supplementary Note Figures
Reference

Simulation pipeline

We simulate the scATAC-seq data according to the method proposed in ref [35] taking bulk ATAC-seq count matrix as input. We take Lun [37] as reference to simulate scRNA-seq data. The detail is described as follows.

(1) Generate the bulk ATAC-seq count matrix.

We generate the bulk ATAC-seq count matrix by a discrete distribution which is calculated from the ENCODE ATAC-seq dataset [36]. We gain a 5,000 by 10 count matrix a_{it}, in which 10 represents 10 cell types (samples) and 5000 is the number of peaks.

(2) Generate the real single cell ATAC-seq data.

We assign the number of cells included in each cell type first and get M cells across all cell types in total. We permute 500 peaks for each cell from the bulk count matrix, generating a 5000 by M single cell count matrix b_{ij}. We generate real single cell ATAC-seq $q_{ij} \in [0,1]$ of a single cell j for the cell type t in peak i as

$$q_{ij} = r_{ij} * N$$

$$r_{ij} = \frac{b_{ij}}{\sum_{i=1}^{M} b_{ij}}$$

Where q_{ij} denotes the probability of single cell j being accessible in peak i, N denotes the number of reads in peaks for each cell.

(3) Generate the observed single cell ATAC-seq data.

We define the observed accessibility $x_{ij} \in \{0,1,2\}$ as

$$x_{ij} \sim Binomial(2, p_{ij})$$

$$p_{ij} = \frac{1}{2} r_{ij} N(1-q) + \frac{1}{2 * 5000} Nq$$

Where p_{ij} defines the probability that a count will be observed in peak i for a single cell, q denotes the noise parameter (0.22 for our study [35]).

Here, we define x_{ij} as a dropout if $x_{ij} = 0$ and $r_{ij} > 0$. The dropout rate α is defined as the number of dropouts divided by the number of nonzero values in r_{ij} matrix.

$$\alpha = \frac{\sum_{i,j} \mathbf{1}_{\{0\}}(x_{ij} r_{ij})}{\sum_{i,j} \mathbf{1}_{(0,+\infty)}(r_{ij})}$$

Where $\mathbf{1}_{\boldsymbol{A}}(\boldsymbol{x}) = \begin{cases} 1, & if\ x \in A \\ 0, & else \end{cases}$.

(4) Simulate cis-regulatory coefficients.

We simulate cis-regulatory coefficient β by sparse normal distribution.

(5) Simulate single cell RNA-seq real data.

We define the real single cell RNA-seq data $C = \left(c_{kj}\right)_{K \times M}$ of the gene k in cell j as

$$C = \beta Q^T$$

(6) Generate the observed single cell RNA-seq data.

To generate a same dropout rate α as ATAC-seq data, we random set the same proportion of C as zero. We denote the new matrix as $\tilde{C}$.

We define the observed gene expression y_{kj} as

$$y_{kj} \sim NB(\widetilde{c_{kj}}, 1/\phi)$$

where ϕ is a dispersion parameter. We set this dispersion parameter as 0.1 according to ref [37].

(7) Generate the unpaired data.

The unpaired data is generated by random selecting half cells from the scRNA-seq data and separating other cells from scATAC-seq data.

Simulation result

According to this procedure, we assign 5 proportions of cell type and generate 5 simulation datasets. For each dataset, we calculate the drop-out rate of ATAC-seq data by cell depth parameter N. The cell type size is listed in the Table S1. In Dataset 1, we assign an equal proportion of cell types. The number of cell types for Dataset 2 follows a negative binomial distribution whose mean is 1000. We set 1-3 minor populations which contain 100 cells for Dataset 3-5.

Dataset	**1**	**2**	**3**	**4**	**5**
cell type 1	1,000	901	100	100	100
cell type 2	1,000	1,070	1,000	100	100
cell type 3	1,000	982	1,000	1,000	100
cell type 4	1,000	1,022	1,000	1,000	1,000
cell type 5	1,000	969	1,000	1,000	1,000
cell type 6	1,000	938	1,000	1,000	1,000
cell type 7	1,000	838	1,000	1,000	1,000
cell type 8	1,000	1,065	1,000	1,000	1,000
cell type 9	1,000	907	1,000	1,000	1,000
cell type 10	1,000	896	1,000	1,000	1,000

Table S1. The number of each cell types of 5 simulation datasets.

Taking the unpaired data as input, we first estimate cis-regulatory coefficients by UnpairReg. Since we know the real cis-regulatory coefficient in this simulation data, we compare the estimated coefficient with the ground truth by calculating Pearson Correlation Coefficients (PCC) to evaluate the coefficient estimation. Figure 2A shows the result of simulation dataset 1 and Fig. N1A (top) denotes the result of simulation dataset 2 to 5. The results suggest that UnpairReg estimates the coefficient accurately.

To evaluate the performance of gene expression prediction, we take the real gene expression data as ground truth and calculate the PCC between our prediction and ground truth. We compare the results with the observed gene expression data, which is affected by dropout. Figure 2B and Fig. N1A (middle) show the average of cell level PCCs of UnpairReg is larger than that of observed gene expression data at each dropout rate. We observe a similar trend in gene level PCCs in Figure 2C and Fig. N1A (bottom).

We also compared the whole distributions of PCCs from UnpairReg with that from observed gene expression data as well as a gene expression prediction based on a random cis-regulatory coefficient at the dropout rate of 0.87 (Figure 2D-E and Fig. N1 B and C).

We observe a remarkable difference between our prediction and the other two predictions of cell level PCC (Figure 2D and Fig. N1B) and gene level PCC (Figure 2E and Fig. N1C) across all simulation datasets.

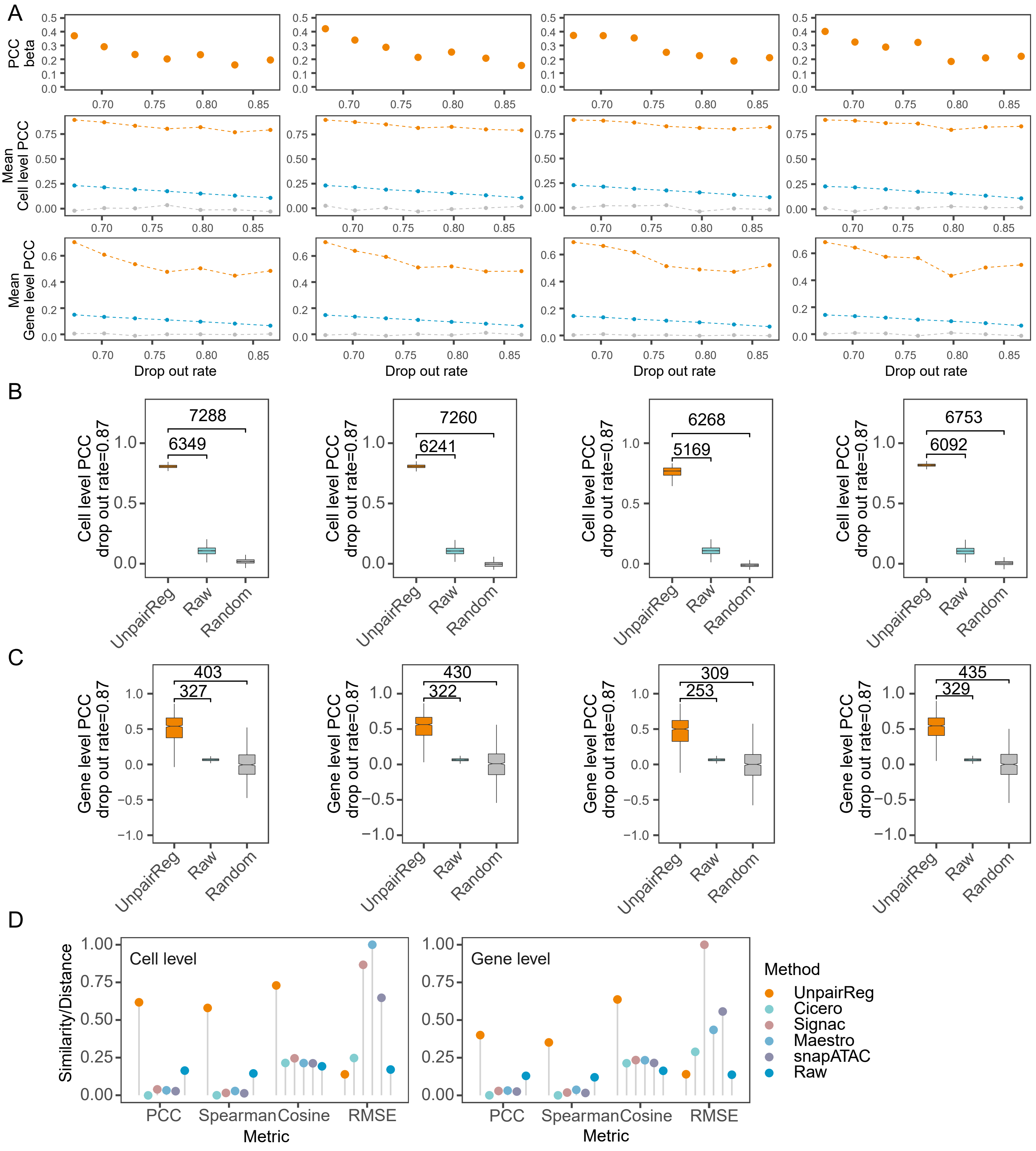

Fig N1. Performance of UnpairReg in silico mixture of cells. (A) The first row is the PCC of UnpairReg predicted coefficient beta and real beta under different dropout rates. The second row is the mean cell level PCCs between predicted gene expression level and real gene expression. The third row is the mean gene level PCCs. The four columns denote four simulation datasets. The first column is the dataset with random cell type size; The second to fourth columns denote the dataset with 1, 2, and 3 minor cell types, respectively. (B) Cell level PCC under the dropout rate of 0.87. (C) The gene level PCCs under the dropout rate of 0.87. (D) The mean gene level and cell level similarity/distance of predicted gene expression and ground truth. RMSE is scaled by dividing by the maximum of six methods. Signac, Cicero, MAESTRO, and snapATAC are four methods to compute the gene activity score. Raw denotes the observed gene expression.

Algorithm performance

Based on simulation data (Dataset 1), we evaluate the UnpairReg algorithm by convergence, initial value performance, sensitivity to the initial value, running time, and memory usage.

(1) Algorithm convergence:

We iterate 100 steps for a UnpairReg initial cis-regulatory coefficient and a random one, respectively, gaining the cost function after each iteration to test the algorithm's convergence. As is shown in Fig. N2A, the cost function monotonously decreases along with the iteration.

(2) Performance of the initial cis-regulatory coefficient:

Fig. N2A shows a huge gap in the cost function in the first iteration between the UnpairReg (9909.2) and random (1839.5) initial cis-regulatory coefficient, which suggests UnpairReg gives a much better initial value than a random one.

(3) Sensitivity of the initial cis-regulatory coefficient:

The cost function of the random case decreases sharply in the first five iterations (from 9909.2 to 1976.5). After the 100th iteration, the cost function of random and UnpairReg initial cases are 1537.3 and 1515.2, respectively, which is close to each other. So UnpairReg is not sensitive to the given initial cis-regulatory coefficient.

(4) Running time

Fixing the number of peaks as 5,000, we test the computational time (running time) under different numbers of genes from 5,000 to 10,000. Fig. N2B shows that running time is linear increasing along with the number of genes. Next, we fix the number of genes as 1,000 and test the running time for different numbers of peaks from 5,000 to 10,000. Running time is linear increasing along with the number of genes (Fig. N2C).

We compare the running time of our algorithm and the quasi-Newton algorithm. We assign 500 peaks, 100 genes, and 10,000 cells, generating simulation data. We perform the UnpairReg and quasi-Newton algorithms, which take 0.25s and 42.94s, respectively, to converge under the same terminate condition.

(5) Memory consumption

We test the memory consumption by the same method with running time. Memory is linear increasing along with the number of genes and peaks (Fig. N2 D and E).

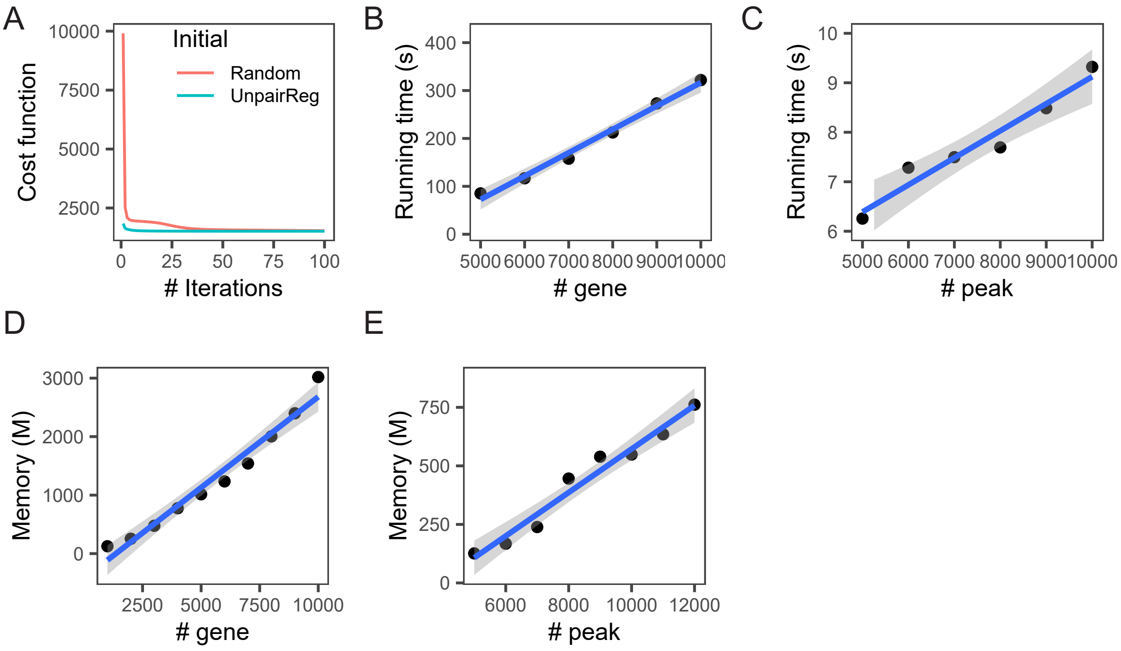

Fig N2. Algorithm performance. (A) Algorithm convergence. The red curve denotes the cost function of the UnpairReg algorithm along with iterations starting with a random initial value. The green curve denotes the cost function starting with the given initial value. (B) Algorithm running time along with the number of genes. The number of peaks is fixed as 5,000. (C) Algorithm running time along with the number of peaks. The number of the gene is fixed as 1,000. (D) Algorithm memory changes along with the number of genes. The number of peaks is fixed as 5,000. (E) Algorithm memory changes over the number of peaks. The number of genes is fixed as 1,000.
