# Supplementary material for: Integration of single-cell multi-omics data by regression analysis on unpaired observations
Source: Genome Biol. 2022 Jul 19;23:160. doi: 10.1186/s13059-022-02726-7 (PMC9295346; doi:10.1186/s13059-022-02726-7)
Supplement: Supplementary file 2 — Additional file 2: Fig S1. UnpairReg gene expression prediction is consistent with the paired data. Fig S2. UnpairReg gene expression prediction of HHBT is consistent with the paired data. Fig S3. Systematical evaluating the performance of UnpairReg. (A) to (C) The mean gene level and cell level similarity/distance of predicted gene expression and raw data. RMSE is scaled by dividing by the maximum of 5 methods. [file 13059_2022_2726_MOESM2_ESM.pdf]

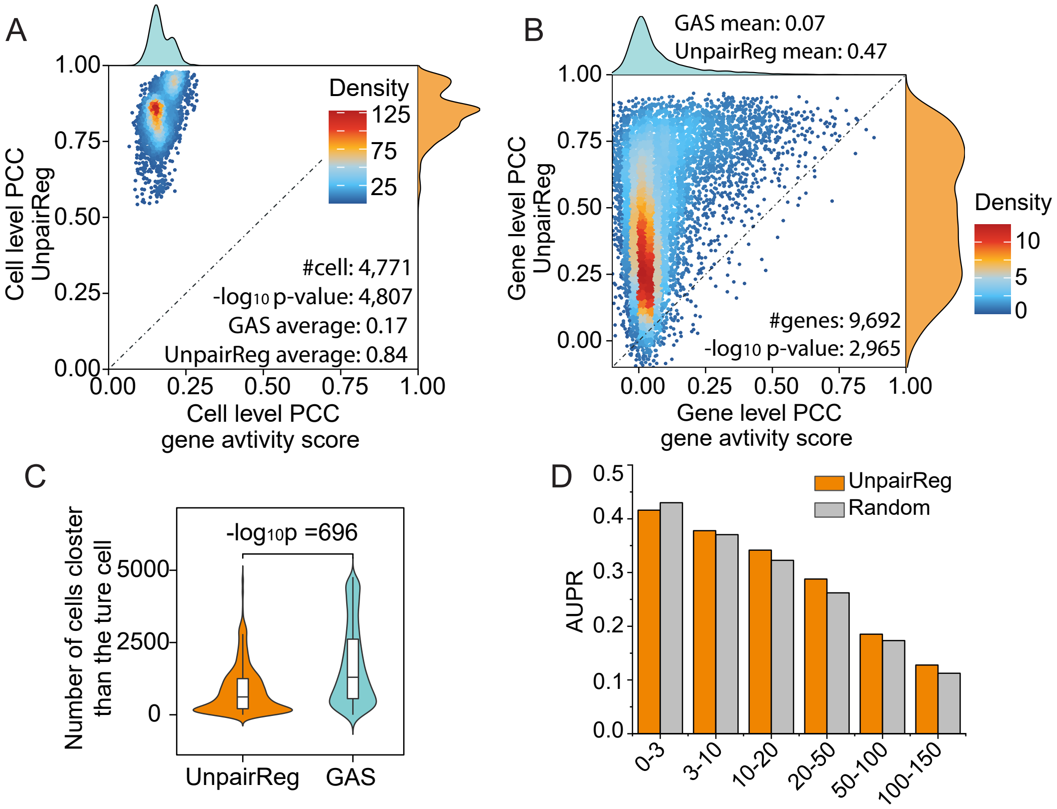

Fig S1. UnpairReg gene expression prediction is consistent with the paired data. (A) Cell level PCC between predicted and imputed gene expression. (B) Gene level PCC between predicted and imputed gene expression. (C) Alignment error of predicted gene expression. For each cell, represented by the predicted gene expression vector, we compute its distance with all cells (impute gene expression). Alignment error for a cell is defined as the number of cells that have a closer distance than the true match (the same cell). (D) The performance metrics AUPR for UnpairReg cis-regulatory coefficients and random values. The ground truth is the variant-gene links from GTEx.

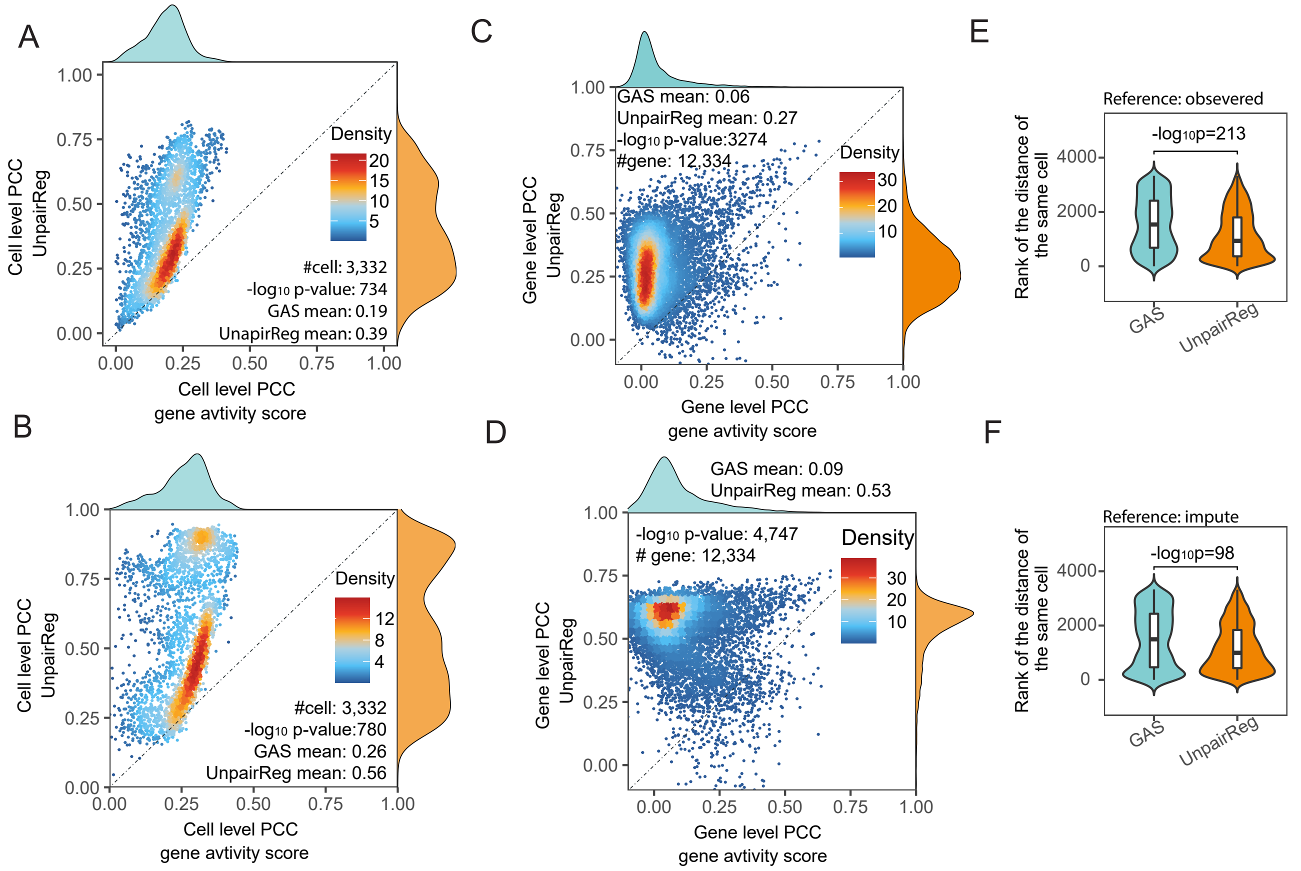

Fig S2. UnpairReg gene expression prediction of HHBT is consistent with the paired data. (A) Cell level PCC between predicted and observed gene expression. (B) Cell level PCC between predicted and impute gene expression. (C) Gene level PCC between predicted and observed gene expression. (D) Gene level PCC between predicted and impute gene expression. (E) Alignment error of predicted gene expression. For each cell, represented by the predicted gene expression vector, we compute its distance with all cells (observed gene expression). (F) Alignment error of predicted gene expression. We compute its distance with all cells for each cell, represented by the predicted gene expression vector (impute gene expression).

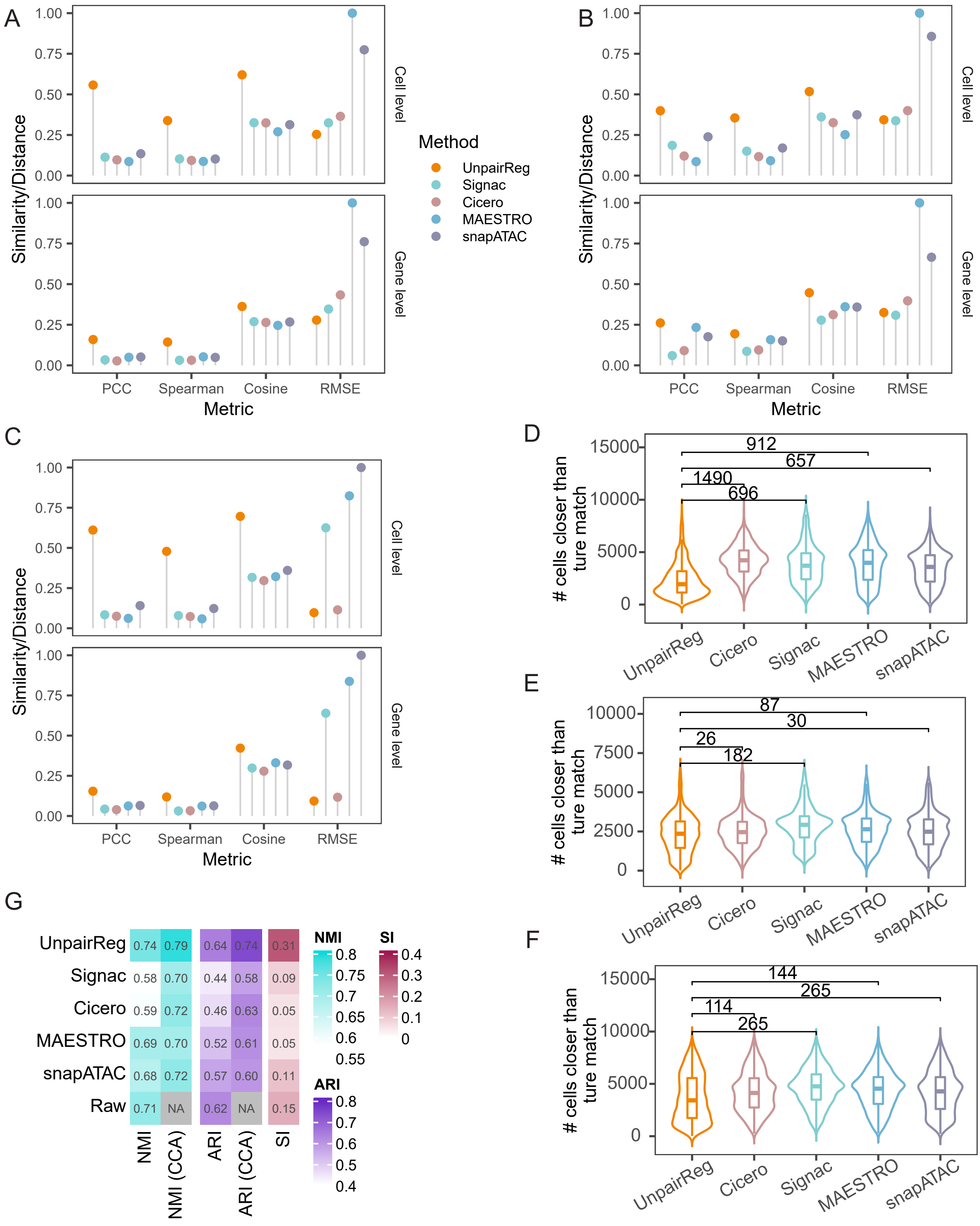

Fig S3. Systematical evaluating the performance of UnpairReg. (A) to (C) The mean gene level and cell level similarity/distance of predicted gene expression and raw data. RMSE is scaled by dividing by the maximum of 5 methods. (A), (B), and (C) correspond to PBMC, HHBT, and EEMB data. (D) to (F) The alignment error of predicted gene expression. We show the log10 p-value of the t-test between UnpairReg and other methods in each figure. (D), (E), and (F) correspond to PBMC, HHBT, and EEMB data. (G) The heatmap of indexes evaluating capacity in identifying the cell types. Normalized Mutual Information (NMI) and Adjusted Rand Index (ARI) denote the clustering results using gene expression prediction data. NMI (CCA) and ARI (CCA) denote clustering results combining the gene expression prediction with the observed gene expression data.
