# Supplementary material for: Integration of single-cell multi-omics data by regression analysis on unpaired observations
Source: Genome Biol. 2022 Jul 19;23:160. doi: 10.1186/s13059-022-02726-7 (PMC9295346; doi:10.1186/s13059-022-02726-7)
Supplement: Supplementary file 3 — Additional file 3.. Review history. [file 13059_2022_2726_MOESM3_ESM.docx]

Review History

**First round of review**

**Reviewer 1**

**Were you able to assess all statistics in the manuscript, including the appropriateness of statistical tests used?**

Yes

**Comments to author:**

In this manuscript authors propose to solve an important problem of pairing regulatory elements with the target genes using unpaired scRNA-seq and scATAC-seq samples. Overall the manuscript is well written, problem is rightly motivated and the solution theoretically makes sense. However I have a few major concerns, one of the major issue the lack of the comparison with other available tools solving problems in the similar space, for example Activity-by-contact model (Fulco, Charles P., et al. "Activity-by-contact model of enhancer-promoter regulation from thousands of CRISPR perturbations." Nature genetics, 2019), a review and benchmark data based on cell-type specific data is also avaialble here (Moore, Jill E., et al. "A curated benchmark of enhancer-gene interactions for evaluating enhancer-target gene prediction methods." Genome biology, 2020). The review mentions multiple methods to connect regulatory elements with target genes. Another useful tool "Baumgarten, Nina, et al. "EpiRegio: analysis and retrieval of regulatory elements linked to genes." Nucleic acids research, 2020.".

In brief, comparison of UnpairReg with just the simplistic model of gene activity score and that too only on the real data (comparison missing on the simulated data) does not justify the usefulness of the proposed algorithm for pairing regulatory elements with the target genes.

Moreover, Seurat itself has a new version of the method which connects scRNA-seq and scATAC-seq data using the multiome kit, (Hao, Yuhan, et al. "Dictionary learning for integrative, multimodal, and scalable single-cell analysis." bioRxiv, 2022) and has shown to perform better for predicting one cellular modality from another, it'd be interesting to check how does UnpairReg compares to that.

Lastly, the tool is written in MATLAB language which requires a paid license to use and hinders the use by a general audience, I myself was not able to use or test the tool as I don't own a license to MATLAB.

Minor Comments:

* I think it's worth showing the coverage plots of some of the connected RE-TG regions which are identified only by UnpairReg and are not predicted significant by GAS.

* In the introduction authors talk about RE (regulatory elements) being millions of base-pairs away from the target genes (TG) being a problem of tools finding RE-TG connection, which is right. However, it has to be made clear that UnpairReg also does not solve this problem; in this study UnpairReg considers RE only within 200kbp near transcript start site, this is not properly clarified in the text.

* There are some typos across the manuscript and it is worth running the manuscript through spell check software, for example Figure 1 the spelling of correlation, spelling of true in the main text is wrong, UpairReg itself is spelled wrong at multiple places.

* Pearson correlation is prone to outliers and it is worth checking the comparisons of Figure 3 on alternative metrics along with PCC, may be spearman correlation or mean absolute relative differences.

* Fig 3E how does GAS (gene actvity score) perform in this metric ?

**Reviewer 2**

**Were you able to assess all statistics in the manuscript, including the appropriateness of statistical tests used?**

Yes

**Comments to author:**

The authors propose a method, named UnpairReg, which uses regression analysis to integrate unpaired single cell multi-omics data. They show examples where they can estimate gene expression from chromatin accessibility data, an example of cell type identification using joint analysis of gene expression and chromatin accessibility data, and an example of inferring a cis-regulatory network. While the overall goal is important and increasingly studied, the evaluation of the method and its comparison to the state-of-the-art are not systematic and, therefore, not convincing. To make general conclusions, more datasets should be analyzed for each task. Instead of separate case studies, a systematic evaluation of the different aspects is needed across all the datasets, always including a comparison to the current state-of-the-art methods as a reference.

Regarding the methods to use in the comparison, currently only the gene activity score is used, which is a very simplistic method, assuming a general correspondence between the chromatin accessibility and gene expression. To provide a more relevant reference, a more advanced approach should be included, such as the recently introduced BABEL, which can be used for unpaired data, once trained on reasonably similar cell types.

Regarding the datasets, it is not clear, how well the simulated dataset represents real datasets. Based on the description an equal proportion of each cell type is assumed and the simulation relies on normal distributions. Are the real datasets alike? Moreover, details of the distributions used are partially missing and should be included.

Overall, the description of the methods lacks details. For instance, it is mentioned that the performance of the gene expression prediction is assessed by alignment error that is described in detail in the Methods. However, no description is provided in the Methods, making it impossible to understand, how it was calculated. E.g. how did you define the distance and how did you deal with the sparsity of the data? Similarly, in Figure 3E, the RE-TG pairs are divided into groups based on their distance but it is not clear, how the distance is defined and how many pairs there are in the different groups. Equations 4 and 5 lack definitions, making them difficult to interpret.

The methods themselves would also require clarification and justification. When it comes to UnpairReg, how well does the iterative algorithm converge? How good is the initial cis-regulatory coefficient estimate? How sensitive is UnpairReg to the initial cis-regulatory coefficient estimate?

Most of the comparisons rely strongly on the Pearson correlation coefficient. However, it is known to be prone to dropouts. How is that dealt with? Are there differences related to this when considering the different methods?

In some comparisons, the real gene expression data is first imputed and the imputed data is then considered that as the ground truth. How is it guaranteed that no bias is introduced with the imputation?

Why one-sample t-test is used to compare between groups?

It is stated that the algorithm is fast, but I did not find any information about what that means in practice. More specific information regarding the computational time and memory consumption is needed.

Finally, the discussion is short and fails to discuss the study in the context of other studies. The following statement in the conclusions seems to go beyond what was studied in this manuscript and should be modified to better reflect the study: "UnpairReg is about 4~5 fold more accurate than the widely used gene activity score, offering a method to obtain all genomics features such as gene expression, chromatin accessibility, DNA methylation, and histone modifications on the same cell."

The language is acceptable for the most part, but there are several typos and, therefore, careful proofreading is needed. However, part of the discussion is impossible to understand and needs to be modified, especially the following: "UnpairReg falls between unsupervised and supervised learning, in which all samples owning features loss label and all samples owning label loss features. Taking the advantage of the input data having multiple response variables and features, our UnpairReg method solves this problem by building using the connection between the covariance matrices of response variables and features."

**Authors Response**

**Point-by-point responses to the reviewers’ comments:**

Reviewer 1:

Comments 1.

In this manuscript authors propose to solve an important problem of pairing regulatory elements with the target genes using unpaired scRNA-seq and scATAC-seq samples. Overall the manuscript is well written, problem is rightly motivated and the solution theoretically makes sense. However I have a few major concerns, one of the major issue the lack of the comparison with other available tools solving problems in the similar space, for example Activity-by-contact model (Fulco, Charles P., et al. "Activity-bycontact model of enhancer-promoter regulation from thousands of CRISPR perturbations." Nature genetics, 2019), a review and benchmark data based on cell-type specific data is also avaialble here (Moore, Jill E., et al. "A curated benchmark of enhancer-gene interactions for evaluating enhancer-target gene prediction methods." Genome biology, 2020). The review mentions multiple methods to connect regulatory elements with target genes. Another useful tool "Baumgarten, Nina, et al. "EpiRegio: analysis and retrieval of regulatory elements linked to genes." Nucleic acids research, 2020.".

*Author’s Response: Thank you for your time and effort in reviewing our manuscript. Please allow us to clarify the motivation for this research first. Many existing scRNA-seq and scATAC-seq data are not paired data, which means they are not measured on the same cell but from the same tissue (Figure R1, left). These types of data are very challenging for computational integrative analysis since these two types of data are neither measured on the same cell nor measuring the same genomics feature. Most of the currently available methods for clustering, pseudotime, trajectory, and visualization are based on a predicted gene expression from chromatin accessibility data (i.e., impute NAs in cells 6 -10 in Figure R1 left). The advantage of this step is to match the features of two types of data to gene level so that many computational techniques can be easily applied. The most commonly used predicted gene expression is called Gene Activity Score (GAS). However, if we take the paired data (Figure R1 right) as ground truth, the gene level PCC of GAS is only about 0.03 (Figure 3B, x-axis). This research aims to improve this prediction without external information (i.e., paired RNA+ATAC data or 3D contact data). To achieve this goal, we developed a regression model on unpaired samples (Figure R1 left) to predict gene expression for cells for which only chromatin accessibility is measured. The results show the accuracy of UnpairReg in gene expression prediction is about 4-5 folds higher than the commonly used methods, and it further improves the downstream analysis like clustering and joint embedding.*

*Figure R1. schematic of Pair and unpair data. RNA-seq data is only available for cells 1-5, and ATAC-seq data is available for cells 6-10. Therefore, traditional methods such as regression model and correlation between peak and gene don’t work for unpair data.*

*Thanks for your comments on linking regulatory elements (REs) and target genes (TGs). We have shown that the regression coefficient from UnpairReg reflects the linkage between RE and TG in Figure 3E. Even though linking RE to TG is not our primary goal, we think it is an interesting question with both biological and methodological significance. In light of your comments, we closely look at these methods in the reviewer’s suggested papers and summarize them in Table R1. From the table, we use the following criteria to choose baseline methods to compare with our method. First, it has to be an unsupervised method rather than a supervised method since our method is an unsupervised method. Supervised methods are expected to perform much better than unsupervised methods. Second, we removed all methods that required paired data since our primary goal is for integrative analysis of unpaired data. Third, we removed some methods using external information like 3D contact data as inputs. Finally, we find only two methods satisfy all three requirements: distance-based method (named Distance in Table R1) and PCC between enhancer and promoter accessibility (named DNase-DNase in Table R1).*

*Table R1. Summary of RE-TG pair prediction methods. Ref denotes the paper mentioned by the reviewer. Pair data denotes whether paired gene expression and chromatin accessibility are required as input.*

*Please see the results of comparison in Figure R2 and the following description in the main text: “To assess the ability of our method to infer cis-regulation, we calculate the consistency of the cis-regulatory coefficients with expression quantitative trait loci (eQTL) studies that link genotype variants to their target genes. We download variant-gene links defined by eQTL in whole blood from GTEx[28] and eQTLGen[29] and use them to validate the RE-TG prediction. As the distance between RE and TG is important for the prediction, we divide RE-TG pairs into different groups based on their distance (0-3kb, 3-10kb, 10-20kb, 20- 50kb, 50-100kb, and 100-150 kb). In each distance group, taking the eQTL data as ground truth, we computed a performance metric, Area Under the Precision-Recall curve (AUPR), by sliding the cisregulatory score. We compared our method with a distance-based method, PCC between enhancer and promoter accessibility, and random predictions. UnpairReg achieves higher AUPR than other methodsin all different distance groups (Fig. 3E, Additional file 1: Fig. S1D). The reason of very low AUPR of PCC in 0- 3k group is that REs in the upstream 2k of TSS are considered as promoters. These results show that UnpairReg not only can accurately predict gene expression but also can provide insight into cis-regulation.”*

*Figure R2. The performance metric AUPR for RE-TG pair prediction methods. eQTL data is from the eQTLGen database.*

Comments 2. In brief, comparison of UnpairReg with just the simplistic model of gene activity score and that too only on the real data (comparison missing on the simulated data) does not justify the usefulness of the proposed algorithm for pairing regulatory elements with the target genes

*Author’s Response: We thank the reviewer’s comment that the comparison is missing on the simulation data. The gene activity score (GAS) from Signac is defined as the number of fragments that falling in the gene body and promoter region. GAS is a method for predicting gene expression rather than a method for linking RE to TG. In the previous manuscript, we used GAS as a baseline method for gene expression prediction and evaluated it by comparing with the real data. In this revision, we add the same comparison for the simulation data. We take real gene expression data from the simulation data as ground truth and evaluate how accurate is the predicted gene expression from UnpairReg. We compare UnpairReg with observed data (Raw) and four other baseline methods, including Signac [4], which is the gene activity score used in our previous manuscript, Cicero [5], MASTRO [6], and snapATAC [7]. We use four metrics to measure the similarity between predicted gene expression and the real gene expression data, which include Pearson’s correlation coefficient (PCC), Spearman’s correlation coefficient (Spearman), cosine similarity (Cosine), and root mean square error (RMSE). RMSE is the lower, the better, and the other three are the higher, the better. Figure R3 shows that UnpairReg performs better than other methods based on all four metrics on simulation data. For detail, please see the Supplementary Note.*

*Figure R3. The gene level and cell level similarity of predicted gene expression and ground truth. RMSE is scaled by dividing by the maximum of six methods.*

Comments 3. Moreover, Seurat itself has a new version of the method which connects scRNA-seq and scATAC-seq data using the multiome kit, (Hao, Yuhan, et al. "Dictionary learning for integrative, multimodal, and scalable single-cell analysis." bioRxiv, 2022) and has shown to perform better for predicting one cellular modality from another, it'd be interesting to check how does UnpairReg compares to that.

*Author’s Response: Thank you for your comment. This paper use multiome data to generate a reference to bridge the unpaired scRNA-seq and scATAC-seq data. Then the query data (scRNA-seq or scATAC-seq) could be mapped into the reference. This method takes multiome data as input, so a direct comparison is unfair for UnpairReg. In addition, the current software package of this new method is not fully available, so we cannot use it for comparison.*

Comments 4. Lastly, the tool is written in MATLAB language which requires a paid license to use and hinders the use by a general audience, I myself was not able to use or test the tool as I don't own a license to MATLAB.

*Author’s Response: Thank you for your valuable comments. In light of your comments, we have implemented our method in R. In the current version, just the basic functions have been implemented, and the speed is slower than the Matlab. In the future, we will use C++ to implement the core function to increase the speed of the R package. The R package is available on the Github at* [*https://github.com/Durenlab/UnpairReg*](https://github.com/Durenlab/UnpairReg)*.*

Minor Comments:

Minor Comments 1. I think it's worth showing the coverage plots of some of the connected RE-TG regions which are identified only by UnpairReg and are not predicted significant by GAS.

*Author’s Response: Thanks for the reviewer’s suggestion. We show the performance of RE-TG pair prediction for one gene as an example. In detail, we use the PCC between RE accessibility and GAS to score the RE-TG pairs. Then we compare this score with the RE-TG pairs score predicted by UnpairReg. Figure R4 shows the performance of RE-TG pairs prediction around gene INO80B by taking the eQTL data as the ground truth.*

*Figure R4. Left: ROC curve of the RE-TG pairs prediction of INO80B by two methods. Right: The color of the first two rows represents the z-score of GAS and UnpairReg, respectively. The third row represents the eQTL data, the red color represents the RE is connected to INO80B in eQTL, and the gray color represents the RE is not connected to the gene in eQTL.*

Minor Comments 2. In the introduction authors talk about RE (regulatory elements) being millions of basepairs away from the target genes (TG) being a problem of tools finding RE-TG connection, which is right. However, it has to be made clear that UnpairReg also does not solve this problem; in this study UnpairReg considers RE only within 200kbp near transcript start site, this is not properly clarified in the text.

*Author’s Response: Thanks for the reviewer’s reminder. The sentence ‘Distance-based linkage is problematic as some REs have connections with TGs from a distance of more than 1 million bp.’ is misleading, so we have removed it.*

Minor Comments 3. There are some typos across the manuscript and it is worth running the manuscript through spell check software, for example Figure 1 the spelling of correlation, spelling of true in the main text is wrong, UnpairReg itself is spelled wrong at multiple places.

*Author’s Response: Thanks for the reviewer’s reminder about the typos across the manuscript. We have checked the manuscript and corrected spelling and grammar mistakes in the manuscript.*

Minor Comments 4. Pearson correlation is prone to outliers and it is worth checking the comparisons of Figure 3 on alternative metrics along with PCC, may be spearman correlation or mean absolute relative differences.

*Author’s Response: For the evaluation metric of gene expression prediction, besides PCC, we added three more metrics (Spearman’s correlation, cosine similarity, and root mean square error) to evaluate our method. Supplementary Figures S3 A, B, and C show the comparison of UnpairReg with four other baseline methods under four different evaluation metrics on three datasets. UnpairReg outperforms other methods under all metrics in all datasets.*

Minor Comments 5. Fig 3E how does GAS (gene activity score) perform in this metric ?

*Author’s Response: To compare the RE-TG prediction of UnpairReg predicted gene and GAS, we calculate TG-RE Pearson’s correlation coefficients between predicted gene expression and chromatin accessibility of RE. We calculate RE-TG PCC of UnpairReg and GAS gene expression prediction separately and obtain the AUPR by taking the eQTL data as ground truth. Figure R5 shows that the AUPR of UnpairReg is higher than that of GAS in the 0-3kb distance group.*

Reviewer #2:

Comments 1. The authors propose a method, named UnpairReg, which uses regression analysis to integrate unpaired single cell multi-omics data. They show examples where they can estimate gene expression from chromatin accessibility data, an example of cell type identification using joint analysis of gene expression and chromatin accessibility data, and an example of inferring a cis-regulatory network. While the overall goal is important and increasingly studied, the evaluation of the method and its comparison to the state-ofthe-art are not systematic and, therefore, not convincing. To make general conclusions, more datasets should be analyzed for each task. Instead of separate case studies, a systematic evaluation of the different aspects is needed across all the datasets, always including a comparison to the current state-of-the-art methods as a reference.

*Author’s Response: We appreciate the reviewer’s comments and suggestions. In light of your comments, we use simulation and real data to systematically evaluate the A) prediction of gene expression, B) mapping of cells, and C) clustering of cells to make general conclusions. We have generated a series of simulation data to validate our method (Please see Supplementary note) systematically. Besides the simulation data, we also tested our method on three different multiome data (PBMC, human brain (HHBT), and mouse brain (EEMB)). In the previous version, we compared our method with Gene Activity Score (Signac). In this revision, we also added three more methods (Cicero [5], MASTRO[6], SnapATAC[7]) for comparison of gene expression prediction. There are two more methods, ArchR[8] and BAP[9], that can predict gene expression from scATAC-seq data. We didn’t include these two methods for comparison because the definitions are very similar to MASTRO, in which gene score is a weighted sum of accessibility of REs and the weight is a decay function of distance to the TSS. Results of systematical evaluation on real data are presented in Supplementary Figure 3.*

*Prediction of gene expression: For the evaluation metric of gene expression prediction, besides PCC, we also added three more metrics (Spearman’s correlation, cosine similarity, and root mean square error) to evaluate our method. Supplementary Figures S3 A, B, and C show the comparison of UnpairReg with four other baseline methods under four different evaluation metrics on three datasets. UnpairReg outperforms other methods under all metrics in all datasets.*

*Mapping of cells: We also compute the alignment error of each cell, which is defined as the number of cells closer than the true match (same cell). For a particular cell, we compute the Euclidian distance between predicted gene expression and the observed gene expression (true match). Then we compute the Euclidian distance between the predicted gene expression of this cell and the observed expression data of other cells. The number of cells with a smaller distance than the true match is defined as an alignment error for this cell (More details in the response for comment 4). Supplementary Figures S3 D, E, and F show that the alignment error of UnpairReg is lower than other methods across the threre datasets.*

*Clustering of cells: To illustrate UnpairReg’s capacity to identify the cell types or subpopulations, we measure whether the cluster structure embodied in the predicted gene expression data agrees with the ground truths cell-type labels. We only use PBMC data to evaluate this because cell label annotation is unavailable for other datasets. First, we take the predicted gene expression as inputs and use Seurat (PCA dimension reduction +shared nearest neighbor + Louvain clustering) to cluster the cells. Then we compare the clustering labels with the ground truth cell label by calculating the Normalized Mutual Information (NMI) and Adjusted Rand Index (ARI) to evaluate the clustering results. UnpairReg achieves NMI 0.74 and ARI 0.64, which are higher than all other methods (Figure R4, the first and the third columns). We also take predicted gene expression and observed gene expression data as inputs and use Seurat V3 (CCA) to cluster the scATAC-seq data with the help of scRNA-seq data. The NMI and ARI of our method are increased to 0.79 and 0.74, which are higher than all other methods (Figure R4, the second and the fourth columns). To quantify the capacity of cell type identification, we calculate the silhouette index (SI) for each cell based on the top 2 to 20 PCs of the gene expression matrix. As a result, all indexes of UnpairReg are higher than the other four methods and the observed gene expression (Raw).*

Comments 2. Regarding the methods to use in the comparison, currently only the gene activity score is used, which is a very simplistic method, assuming a general correspondence between the chromatin accessibility and gene expression. To provide a more relevant reference, a more advanced approach should be included, such as the recently introduced BABEL, which can be used for unpaired data, once trained on reasonably similar cell types

*Author’s Response: In this revision, we have compared our method with several other methods (Please see the response to comment 1). The BABEL is a fantastic method for the integration of scRNA-seq and scATAC-seq data. However, it takes paired scRNA-seq and scATAC-seq data, which means the gene expression and chromatin accessibility must be measured on the same cell. Here our method takes unpaired data as inputs, which means scRNA-seq and scATAC-seq are not profiled from the same cell, but from the same heterogeneous population. To avoid misunderstanding, we explained the input of our method and emphasized the difference with paired data in the introduction. Based on the BABEL paper, BABEL achieves a Pearson correlation of 0.62 and Spearman’s correlation of 0.35 when predicting RNA expression from ATAC accessibility (Figure 2 B-C of the BABEL paper). Note that this correlation is different from our cell-level correlation and gene-level correlation. They reshape the whole predicted gene expression matrix into a long vector and calculate the correlation. In PBMC data, such Pearson and Spearman correlation of UnpairReg are 0.54 and 0.32, respectively. UnpairReg achieves a slightly lower accuracy than BABEL, which should be considered as a very good result. We understand that this is not a rigorous comparison since input data are not exactly the same. We didn’t further compare our method with BABEL since they require different types of inputs.*

Comments 3. Regarding the datasets, it is not clear, how well the simulated dataset represents real datasets. Based on the description an equal proportion of each cell type is assumed and the simulation relies on normal distributions. Are the real datasets alike? Moreover, details of the distributions used are partially missing and should be included.

*Author’s Response: We thank the reviewer’s advice to improve the simulation to represent real datasets better. In this revision, we removed the normal distribution assumption and generated a series of simulation data with different proportions of cell types. We simulate the scATAC-seq data according to the method proposed in ref [10] by taking a bulk ATAC-seq count matrix as input. We use Lun[11] to simulate scRNAseq data. The detail is described in the Supplementary Note.*

Comments 4. Overall, the description of the methods lacks details. For instance, it is mentioned that the performance of the gene expression prediction is assessed by alignment error that is described in detail in the Methods. However, no description is provided in the Methods, making it impossible to understand, how it was calculated. E.g. how did you define the distance and how did you deal with the sparsity of the data? Similarly, in Figure 3E, the RE-TG pairs are divided into groups based on their distance but it is not clear, how the distance is defined and how many pairs there are in the different groups. Equations 4 and 5 lack definitions, making them difficult to interpret.

*Author’s Response: Thanks for the reviewer’s comments. We modified these problems one by one. (1) We add the details of the alignment error in the Methods as follows. Alignment error is used to evaluate the alignment accuracy of each cell. Denote X and Y as the predicted gene expression matrix and ground truth of gene expression. The alignment error for the 𝑘𝑘 th cell is defined as:*

*Where* *is the number of cells which is closer to the kth cell than the same cell in the ground truth gene expression data, 𝑑 is the Euclidiean distance xk and 𝑦𝑘. represent the predicted and ground truth gene expression of the kth cell, respectively.*

*(2) We add explanations of the distance and the number of pairs in different groups in the figure legend. We divide RE-TG pairs into different groups based on the physical distance (how many base pairs) from the location of RE and the TSS of TG. There are 8634, 5791, 7431, 21282, 33205, and 30850 RE-TG pairs in 0-3k bp, 3-10k bp, 10-20k bp, 20-50k bp, 50-100k bp,100-150k bp groups respectively.*

*(3) We complete definitions of equation 4 as follows.*

*We have gained the predicted gene expression from the algorithm above based on the gene-gene covariance and RE-RE covariance. To improve the gene expression in preserving both cell-cell covariance and gene-gene covariance better, we developed an optimization model to fine tune the predicted gene expression further. We design an optimization model to find a fine-tuned gene expression X, so that the predicted gene-gene covariance (XtX) is same with the real gene-gene covariance (𝑅1) and meanwhile the predicted RE-RE covariance (𝑋Xt) is also same with the real one:*

*Here,**aims to preserve gene-gene covariance, where**is the gene-gene covariance, defined by**timing a scaling factor, the ratio of the number of cells, to eliminate the gap of cell number between RNA-seq and ATAC-seq, matching the scale of EtE and XtX.* *aims to preserve cell-cell covariance, where**is the cell-cell covariance, defined by OOt timing another scaling factor the ratio of the mean of EEt and OOt to eliminate the difference of data type. 𝜆 is a tuning parameter to weight the two objectives. The scaling factor is introduced. We design a fixed point iterative algorithm, taking**as the start point of X, renewing 𝑋𝑋 by* *where*

Comments 5. The methods themselves would also require clarification and justification. When it comes to UnpairReg, how well does the iterative algorithm converge? How good is the initial cis-regulatory coefficient estimate? How sensitive is UnpairReg to the initial cis-regulatory coefficient estimate?

*Author’s Response: Thanks for the reviewer’s reminder for the description of the algorithm. We use simulation Dataset 1 described in the supplementary note, for examples to test the performance of our algorithm. To test the algorithm's convergence, we start with the default initial of 𝛽𝛽 and integrate 100 steps. Supplementary Note Fig N2A shows that the cost function is monotonically decreased along with the iteration. If we start with a random initial, the initial cost is about 5-fold higher than the initial cost of the default initial. However, the cost decreases dramatically in 5 interactions (from 9909.2 to 1976.5) and converges to a similar level to the cost that starts with the default initial (1537.3 and 1515.2, respectively). These results show that UnpairReg gives a much better default initial value than a random one. Thus, we conclude that our default initial decreases the number of iterations to convergence, and the algorithm of UnpairReg is not sensitive to the given initial cis-regulatory coefficient. This part was described in the Supplementary Note.*

Comments 6. Most of the comparisons rely strongly on the Pearson correlation coefficient. However, it is known to be prone to dropouts. How is that dealt with? Are there differences related to this when considering the different methods?

*Author’s Response: We totally agree with the reviewer, and we add three different metrics for comparison. The result is discussed in response to comment 1.*

Comments 7. In some comparisons, the real gene expression data is first imputed, and the imputed data is then considered that as the ground truth. How is it guaranteed that no bias is introduced with the imputation?

*Author’s Response: We agree with the reviewer that the imputation is not the same as real gene expression data. In the paper, we use both raw and imputation data as ground truth. The main conclusion of this section is that UnapirReg predicted gene expression is more accurate than other baseline methods. This conclusion is based on the comparison that takes the raw data as ground truth. However, using raw data as ground truth would underestimate the accuracy (i.e., PCC) as the data is affected by drop-outs. To provide a reasonable estimation of the accuracy, we also use the imputed data as ground truth and calculate PCC. We agree that the imputation may induces bias. Thus, the real accuracy should be between the range of raw data-based PCC and imputed data-based PCC. We have added discussion in the main text.*

Comments 8. Why one-sample t-test is used to compare between groups?

*Author’s Response: We could use a two-sample t-test to compare between groups. However, we think a one-sample test (paired-test) is more sensitive for this case since the samples in the two group are identical. For example, when we test gene-level PCC, we have 20k genes’ PCC in each group. Two sample t-test is just testing if the whole distribution is shifted, while the one-sample t-test considers the pairing information (i.e., PCC of gene Foxa1 in group 1 is higher than PCC in group 2). We are testing whether the mean of the difference of PCC between the two groups over all genes is different from zero.*

Comments 9. It is stated that the algorithm is fast, but I did not find any information about what that means in practice. More specific information regarding the computational time and memory consumption is needed.

*Author’s Response: We thank the reviewer for pointing out this question. We test the computational time and memory based on the simulation data. Please see the Algorithm performance section in the Supplementary Note.*

Comments 10. Finally, the discussion is short and fails to discuss the study in the context of other studies. The following statement in the conclusions seems to go beyond what was studied in this manuscript and should be modified to better reflect the study: "UnpairReg is about 4~5 fold more accurate than the widely used gene activity score, offering a method to obtain all genomics features such as gene expression, chromatin accessibility, DNA methylation, and histone modifications on the same cell."

*Author’s Response: We have revised the statement as follows: “Taking the single cell RNA-seq data as ground truth, we compare the mean cell level PCC and gene level PCC of UnpairReg and other four methods across three datasets. The predicted gene expression from UnpairReg is about 1.67-9.93 fold (cell level PCC) and 1.12-5.78 fold (gene level PCC) more accurate than the widely used gene activity score, offering a method to obtain gene expression and chromatin accessibility on the same cell.”*

Comments 11. The language is acceptable for the most part, but there are several typos and, therefore, careful proofreading is needed. However, part of the discussion is impossible to understand and needs to be modified, especially the following: "UnpairReg falls between unsupervised and supervised learning, in which all samples owning features loss label and all samples owning label loss features. Taking the advantage of the input data having multiple response variables and features, our UnpairReg method solves this problem by building using the connection between the covariance matrices of response variables and features."

*Author’s Response: We thank you for your thorough advice. We have carefully proofread the paper and corrected several typos and grammar issues. We have modified the statement as follows:*

*"From the machine learning point of view, the linear regression model using paired data to predict gene expression belongs to supervised learning, in which the feature is chromatin accessibility, and the label is gene expression. For supervised learning, features and labels are accessible for each sample. Current gene expression estimation methods, such as gene activity score, only use the chromatin accessibility data. These methods belong to unsupervised learning, in which the data is not labelled. UnpairReg falls between unsupervised and supervised learning. In detail, for each cell (sample) from scATAC-seq data, chromatin accessibility (feature) is accessible, but gene expression (label) is not accessible. For each sample in single cell RNA-seq data, the label is accessible, but the feature is not. Taking the advantage of the input data having multiple response variables and features, UnpairReg solves this problem by building the connection between the covariance matrices of response variables and features.”*

**Second round of review**

**Reviewer 1**

Authors have satisfactorily addressed all my comments and I'd like to Congratulate the authors for the great work !

**Reviewer 2**

The authors have addressed some of my comments, but my major concerns remain. In particular, although more comparisons are now provided against selected methods, no information is provided on how they were actually applied (e.g. which version, how were batch effects and dropouts dealt with, etc.). There also seems to be complete ignorance regarding available tools for single-cell multi-omics integration of unpaired multi-omics data. In addition, I would again recommend careful proofreading, as there are still several typos in the manuscript.

**Authors Response**

**Point-by-point responses to the reviewers’ comments:**

*Author response: We provided details about methods that were compared, including the version of package, function, and website of pipelines. We added a subsection, Model parameters (in page 12), in Methods section of the manuscript.*
